# EIF4A3‐Induced Circular RNA CircDdb1 Promotes Muscle Atrophy through Encoding a Novel Protein CircDdb1‐867aa

**DOI:** 10.1002/advs.202406986

**Published:** 2024-10-16

**Authors:** Xiaolan Zhu, Tingting Yang, Yongjun Zheng, Qiumeng Nie, Jingying Chen, Qian Li, Xinyi Ren, Xiaohang Yin, Siqi Wang, Yuwei Yan, Zhengyu Liu, Ming Wu, Dongchao Lu, Yan Yu, Lei Chen, Emeli Chatterjee, Guoping Li, Dragos Cretoiu, T Scott Bowen, Jin Li, Junjie Xiao

**Affiliations:** ^1^ Cardiac Regeneration and Ageing Lab Institute of Geriatrics (Shanghai University) Affiliated Nantong Hospital of Shanghai University (The Sixth People's Hospital of Nantong) and School of Life Sciences Shanghai University Nantong 226011 China; ^2^ Institute of Cardiovascular Sciences Shanghai Engineering Research Center of Organ Repair Joint International Research Laboratory of Biomaterials and Biotechnology in Organ Repair (Ministry of Education) School of Life Sciences Shanghai University Shanghai 200444 China; ^3^ Division of Pain Management Huadong Hospital Affiliated to Fudan University Shanghai 200040 China; ^4^ Department of Orthopedics Shanghai Gongli Hospital Shanghai 200135 China; ^5^ School of Integrative Medicine Shanghai University of Traditional Chinese Medicine Shanghai 201203 China; ^6^ Department of Spine Surgery Tongji Hospital School of Medicine Tongji University Shanghai 200065 China; ^7^ Cardiovascular Division of the Massachusetts General Hospital and Harvard Medical School Boston MA 02114 USA; ^8^ Department of Medical Genetics Carol Davila University of Medicine and Pharmacy Bucharest 020031 Romania; ^9^ Materno‐Fetal Assistance Excellence Unit Alessandrescu‐Rusescu National Institute for Mother and Child Health Bucharest 011062 Romania; ^10^ School of Biomedical Sciences Faculty of Biological Sciences University of Leeds Leeds LS2 9JT UK

**Keywords:** circDdb1, eEF2, muscle aging, muscle atrophy, translation

## Abstract

Little is known about if and how circular RNAs (circRNAs) are involved in skeletal muscle atrophy. Here a conserved circular RNA Damage‐specific DNA binding protein 1 (circDdb1), derived from the host gene encoding Damage‐specific DNA binding protein 1 (DDB1), as a mechanism of muscle atrophy is identified. circDdb1 expression is markedly increased in a variety of muscle atrophy types in vivo and in vitro, and human aging muscle. Both in vivo and in vitro, ectopic expression of circDdb1 causes muscle atrophy. In contrast, multiple forms of muscle atrophy caused by dexamethasone, tumor necrosis factor‐alpha (TNF‐α), or angiotensin II (Ang II) in myotube cells, as well as by denervation, angiotensin II, and immobility in mice, are prevented by circDdb1 inhibition. Eukaryotic initiation factor 4A3 (EIF4A3) is identified as a regulator of circDdb1 expression in muscle atrophy, whereas circDdb1 encodes a novel protein, circDdb1‐867aa. circDdb1‐867aa binds with and increases the phosphorylation level of eukaryotic elongation factor 2 (eEF2) at Thr56 to reduce protein translation and promote muscle atrophy. In summary, these findings establish circDdb1 as a shared regulator of muscle atrophy across multiple diseases and a potential therapeutic target.

## Graphical abstract

circDdb1 is increased in muscle atrophy models and aged muscle. Overexpression of circDdb1 promotes muscle atrophy, while knockdown of circDdb1 attenuates that. circDdb1 promotes muscle atrophy by encoding a novel protein circDdb1‐867aa. circDdb1‐867aa binds with and increases the phosphorylation level of eukaryotic elongation factor 2 (eEF2) at Thr56 to reduce protein translation and promote muscle atrophy. Collectively, these findings establish circDdb1 as a shared regulator of muscle atrophy across multiple diseases and a potential therapeutic target.

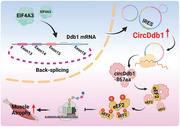

## 1 Introduction

Loss of muscle mass and dysfunction are the hallmarks of skeletal muscle atrophy, which raises the risk of morbidity and death and lowers quality of life.^[^ ^1^ ^]^ Muscle atrophy is associated with aging, starvation, prolonged disuse as well as many diseases.^[^ ^2^ ^]^ The intrinsic regulatory mechanism of muscle atrophy is intricate.^[^ ^3^ ^]^ Typically, the autophagy‐lysosome and ubiquitin‐proteasome pathways are triggered in muscle atrophy.^[^ ^4^ ^]^ MAFbx/atrogin‐1 and MuRF1, two E3‐ubiquitin ligases, are considered molecular markers of muscle atrophy and exhibit elevated levels in nearly all types of muscle wasting.^[^ ^5^ ^]^ Furthermore, the skeletal muscle protein synthesis pathway is intimately linked to the suppressed mammalian target of rapamycin (mTOR) signaling pathway.^[^ ^6^ ^]^ But as of right now, there are no effective medications to treat muscle atrophy. Thus, it is imperative to investigate the underlying processes of muscle atrophy in more detail and to develop novel treatment approaches.

Circular RNAs (circRNAs), which are distinctive for their single‐stranded, covalently closed structure formed by the back‐splicing of pre‐mRNAs, are impervious to exonuclease cleavage due to their continuous loop configuration. Consequently, these RNAs exhibit greater stability and a prolonged half‐life in comparison to linear RNAs.^[^ ^7^ ^]^ Circular RNAs have a role in several physiological and pathological processes. According to recent findings, circRNAs primarily function as molecular sponges for miRNAs, interacting with RNA‐binding proteins, regulating transcription, competitively inhibiting mRNA precursors, and translating peptides or proteins.^[^ ^8^ ^]^

In skeletal muscle tissue, certain circRNAs have been identified to participate in the process of myogenesis. For instance, circSNX29 functions as a molecular sponge for miR‐744, thereby activating the Wnt5a/Ca^2+^ pathway and regulating myoblast proliferation and differentiation.^[^ ^9^ ^]^ Likewise, studies have shown that circHIPK3,^[^ ^10^ ^]^ circINSR,^[^ ^11^ ^]^ circFGFR2,^[^ ^12^ ^]^ and circUSP13^[^ ^13^ ^]^ function as molecular sponges for various miRNAs, thereby modulating myoblast proliferation and differentiation. In line with this, it has been reported that circ‐ZNF609 can translate specific polypeptides to promote myogenesis.^[^ ^14^ ^]^ We also recently identified circTmeff1 as a common circRNA regulator and novel therapeutic target for multiple types of muscle atrophy.^[^ ^15^ ^]^ However, more circRNAs involved in the regulation of muscle atrophy likely remain unidentified.

In this study, the conserved circRNA circDdb1, which is generated from the host gene encoding the Damage‐specific DNA binding protein 1 (DDB1), was identified as a shared regulator and a useful target for therapeutic intervention in muscle atrophy. circDdb1 was significantly upregulated in multiple types of muscle atrophy in vivo and in vitro, and human aging muscle. Muscle atrophy could be induced by overexpressing circDdb1, while different forms of muscle atrophy can be prevented by repressing circDdb1. We further identified that eukaryotic initiation factor 4A3 (EIF4A3) induced the circularization of circDdb1 in muscle atrophy by binding with its upstream flanking sequence. In addition, circDdb1 promoted muscle atrophy by encoding a novel protein circDdb1‐867aa. Taken together, we discovered circDdb1 as a promising target for the prevention of multiple types of muscle atrophy.

## 2 Results

### 2.1 CircDdb1 Is Increased in Multiple Muscle Atrophy Conditions

**Figure 1:**
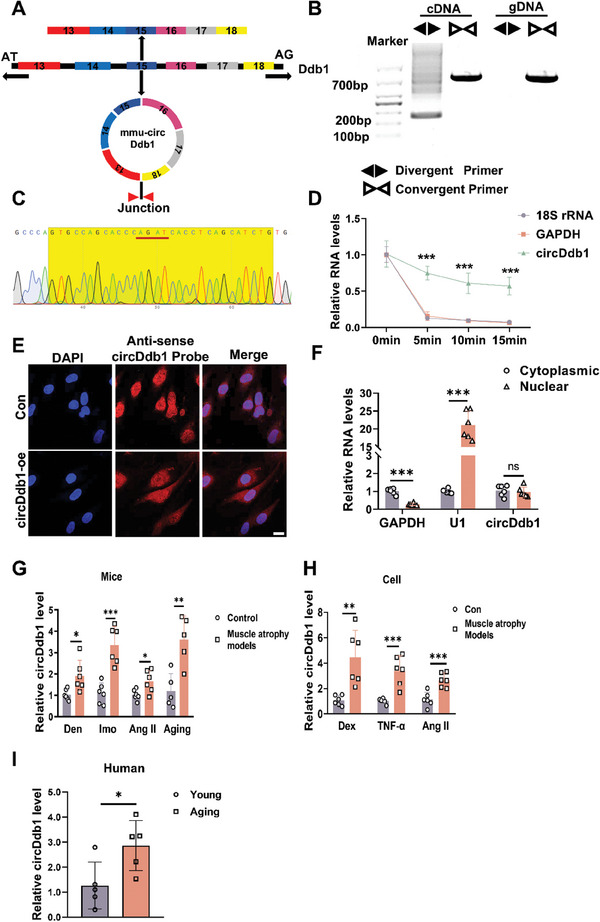
Circular RNA circDdb1 was upregulated in muscle atrophy. A) The circDdb1 structure. B) PCR confirmed the back splicing sequences and representative agarose gel electrophoresis images. C) Sanger sequencing analysis of the splicing junction site of circDdb1. D) qRT‐PCR assessing circDdb1 expression level in C2C12 myotubes treated with RNase R for 5min, 10min, and 15min (*n* = 4). 18S rRNA and GAPDH mRNA were used as negative controls. E) Representative images of FISH showing the distribution of circDdb1 in C2C12 myoblast cells (scale bar: 10 µm). F) qRT‐PCR assessing the expression of circDdb1 in the nucleus and cytoplasm of C2C12 myotubes (*n* = 6). G) qRT‐PCR assessing circDdb1 expression level in animal muscle atrophy models (including Den‐, Imo‐, and AngII‐induced muscle atrophy models) (*n* = 6). H) qRT‐PCR assessing circDdb1 expression level in cell muscle atrophy models (including Dex, TNF‐α, and AngII‐treated C2C12 myotubes) (*n* = 6). I) qRT‐PCR assessing circDdb1 expression level in young and aged men and women muscle lysates (*n* = 5). Statistical analysis was performed using an unpaired, two‐tailed Student's *t*‐test to compare between two groups. ^*^ *p* < 0.05; ^**^ *p* < 0.01; ^***^ *p* < 0.001. Data are represented as mean ± SD.

In order to discover the functional circRNAs associated with muscle atrophy, we performed circRNA‐seq in mice with and without denervation‐induced muscle atrophy.^[^ ^15^ ^]^ Mmu_circ_0007604 located in mouse chr19:10696030‐10698128, and back‐spliced from exons 13–18 of the transcript of DDB1 gene with a length of 867 nucleotides (thus termed as circDdb1) (**Figure** **1**A). Using the circBank database set and sequence alignment, we found that circDdb1 was highly conserved between humans (circBase ID: hsa_circ_0022284; Position: chr11: 61079255–61081958 strand:‐) and mice (Figure S1, Supporting Information). CircDdb1 was upregulated in the denervation‐induced muscle atrophy model compared to sham control according to our previously published study^[^ ^15^ ^]^ (Figure S2A, Supporting Information). We first designed circDdb1‐specific convergent and divergent primers and validated these primers in C2C12 myotube cells. We observed the expected PCR product with the correct size of circDdb1, which amplified with the convergent primer, in cDNA and gDNA. In addition, the divergent primers can only amplify circDdb1 using cDNAs as a template, and no PCR product was detected in gDNA (Figure 1B). Following with sanger sequence of PCR product amplified with divergent primer, we confirmed the correct back splicing region of circDdb1 (Figure 1C). Furthermore, the circularization structure of circDdb1 was verified by performing an RNase R treatment on the RNA extracted from C2C12 cells. We observed that circDdb1 can resist RNase R cleavage compared to linear GAPDH mRNA and 18S rRNA in C2C12 cells (Figure 1D). Moreover, the sub‐cellular fractionation assay and RNA FISH analysis revealed that circDdb1distributed in the cytoplasm and nuclear both in myoblast and myotube (Figure 1E,F; Figure S2B, Supporting Information).

We used commonly used in vivo and in vitro muscle atrophy models to investigate the expression of circDdb1 during muscle atrophy in more detail. The increased levels of circDdb1 were observed in TNF‐α, Dexamethasone (Dex), Angiotensin II (AngII)‐induced atrophy in vitro cell models, and a range of in vivo mice models of muscle atrophy, including denervation (Den), immobilization (Imo), AngII, and aging (Figure 1G,H). In particular, circDdb1 was also increased in aged human muscles (Figure 1I; Figure S2C, Supporting Information). In conclusion, our findings imply that muscle atrophy is linked to circDdb1.

### 2.2 CircDdb1 Is a Pro‐Atrophic Factor

**Figure 2:**
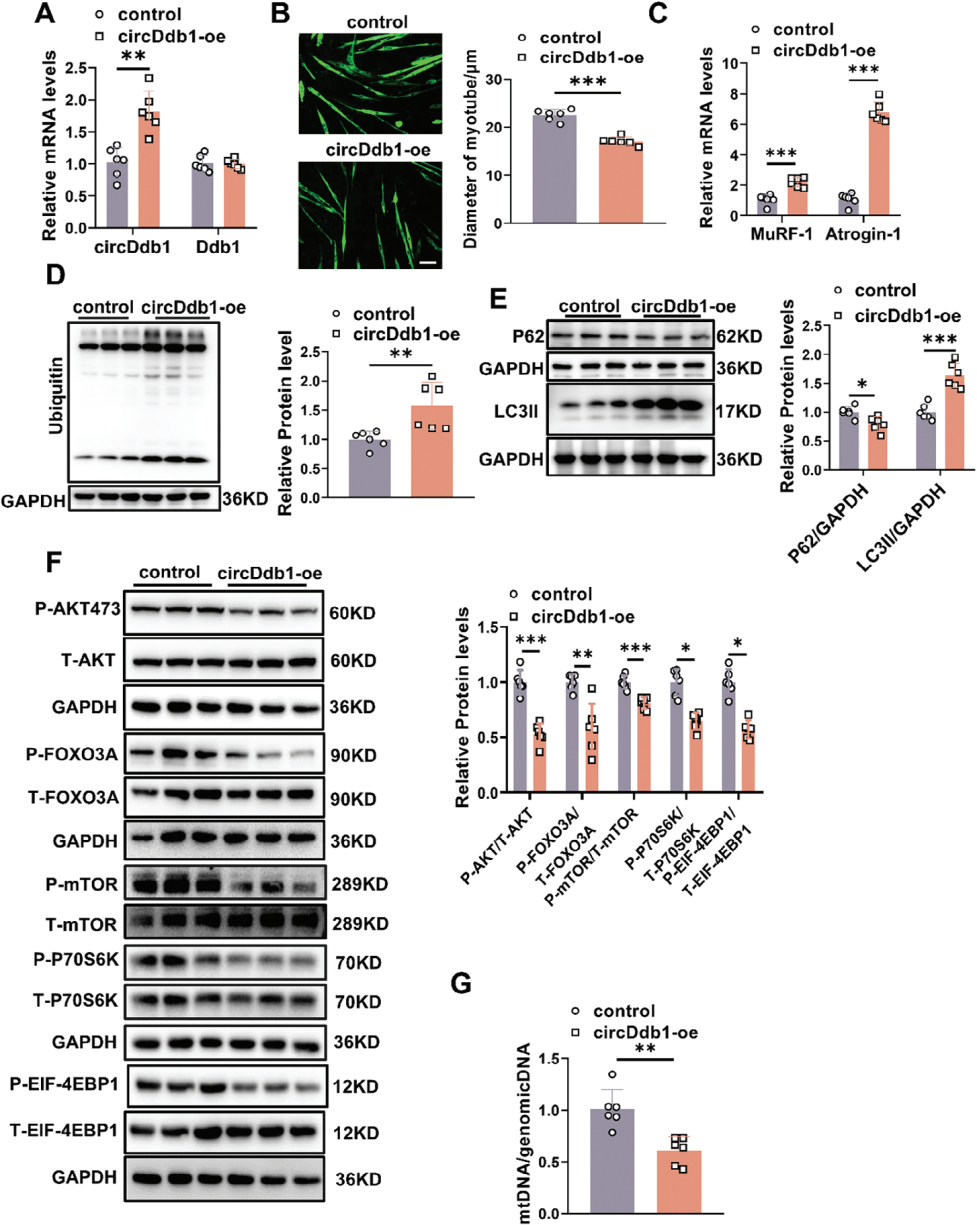
circDdb1 triggers muscle atrophy in vitro. A) qRT‐PCR assessing circDdb1 and Ddb1 expression levels in C2C12 myotubes transfected with circDdb1‐oe and control (*n* = 6). B) Representative images and statistical analysis of the diameter of C2C12 myotubes in C2C12 myotubes transfected with circDdb1‐oe and control (*n* = 6), scale bar: 100 µm. C) qRT‐PCR assessing MuRF‐1 and Atrogin‐1 gene expression levels in C2C12 myotubes transfected with circDdb1‐oe and control (*n* = 6). D) Western blot assessing Ubiquitin protein expression level in C2C12 myotubes transfected with circDdb1‐oe and control (*n* = 6). E) Western blot assessing P62 and LC3 protein expression levels in C2C12 myotubes transfected with circDdb1‐oe and control (*n* = 6). F. Western blot assessing AKT/FOXO3A/mTOR pathway‐related protein expression levels (including p‐AKT, p‐FOXO3A, p‐mTOR, p‐P70S6K, p‐EIF‐4EBP1) in C2C12 myotubes transfected with circDdb1‐oe and control (*n* = 6). G) qRT‐PCR assessing the mt‐Col1 expression in C2C12 myotubes transfected with circDdb1‐oe and control (*n* = 6). Statistical analysis was performed using an unpaired, two‐tailed Student's *t*‐test to compare between two groups. Data are represented as mean ± SD. ^*^ *p* < 0.05; ^**^ *p* < 0.01; ^***^ *p* < 0.001. circDdb1‐oe: circDdb1 overexpression plasmid; control: control plasmid.

In completely differentiated C2C12 myotubes, circDdb1 was overexpressed to investigate the possible involvement of circDdb1 in muscle atrophy. Overexpression of circDdb1 efficiently increased circDdb1 in myotube cells, without changing the expression of linear Ddb1 (**Figure** **2**A). CircDdb1 overexpression led to myotube atrophy, as assessed by immunofluorescence (IF) staining for myosin heavy chain (MHC) to quantify changes in myotube size and by increased mRNA expression of the established pro‐atrophic markers, Atrogin‐1 and MuRF‐1 mRNA (Figure 2B,C). Accordingly, circDdb1 overexpression increased indicators of the autophagy‐lysosome pathway (ALP) and the ubiquitin‐proteasome system (UPS) in myotube cells relative to the control group, as shown by western blot and qRT‐PCR for the examination of associated gene and protein expression (Figure 2D,E; Figure S3A,B, Supporting Information). However, cell apoptosis was not affected by circDdb1 overexpression (Figure S3C, Supporting Information). In addition, circDdb1 suppressed the protein synthesis‐associated AKT/FOXO3A/mTOR signaling pathway, as seen by a reduction in the phosphorylation of mTOR, P70S6K, 4EBP1, AKT (Ser‐473), and FOXO3A (Ser‐253) (Figure 2F). Besides, circDdb1 overexpression showed significantly decreased mitochondria, as indicated by the mtDNA copy numbers (Figure 2G). These data indicated that circDdb1 was a pro‐atrophy factor in vitro.

**Figure 3:**
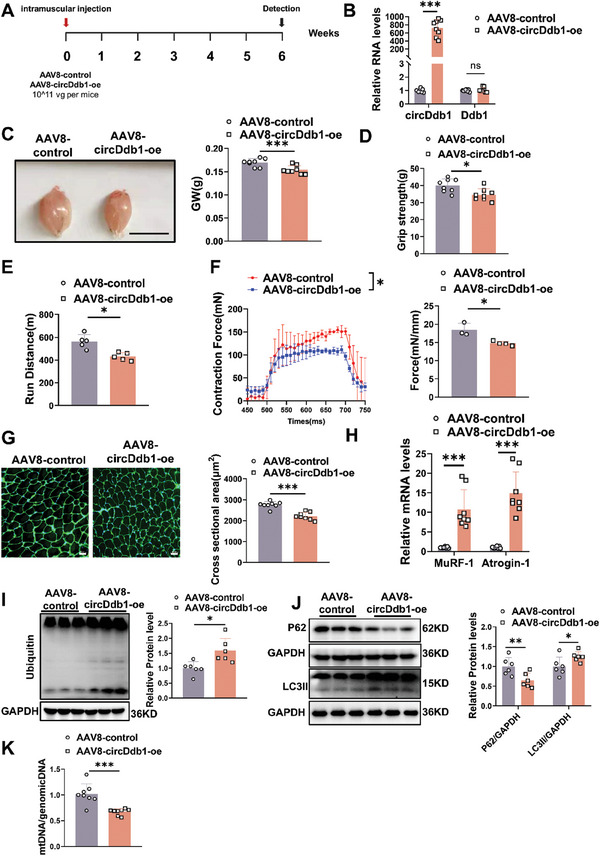
circDdb1 induces muscle atrophy in vivo. A) Schematic representation of the experimental design for viral injection. B) qRT‐PCR assessing circDdb1 expression level in gastrocnemius muscle of mice injected with AAV8‐circDdb1‐oe and AAV8‐control (*n* = 8). C) Representative images of gastrocnemius muscle morphology and gastrocnemius muscle weight (GW) of mice injected with AAV8‐circDdb1‐oe and AAV8‐control (*n* = 8, scale bar: 1 cm). D) Statistical analysis of grip strength in the right hind limb muscles of mice injected with AAV8‐circDdb1‐oe and AAV8‐control (*n* = 8). E) The running distance of mice that were injected with AAV8‐circDdb1‐oe and AAV8‐control (*n* = 5). F) Statistical analysis of EDL muscle contraction force of mice injected with AAV8‐circDdb1‐oe and AAV8‐control (*n* = 3–4). G) Representative images and statistical analysis of myofiber cross‐sectional area (CSA) of mice injected with AAV8‐circDdb1‐oe and AAV8‐control (*n* = 8; scale bar: 50 µm). H) qRT‐PCR assessing MuRF‐1 and Atrogin‐1 gene expression levels in gastrocnemius muscle of mice injected with AAV8‐circDdb1‐oe and AAV8‐control (*n* = 8). I) Western blot assessing Ubiquitin protein expression level in gastrocnemius muscle of mice injected with AAV8‐circDdb1‐oe and AAV8‐control (*n* = 6). J) Western blot assessing P62 and LC3 protein expression levels in gastrocnemius muscle of mice injected with AAV8‐circDdb1‐oe and AAV8‐control (*n* = 6). K) qRT‐PCR assessing the mt‐Col1 in gastrocnemius muscle of mice injected with AAV8‐circDdb1‐oe and AAV8‐control (*n* = 8). Statistical analysis was performed using an unpaired, two‐tailed Student's t‐test to compare between two groups. Data are represented as mean ± SD. ^*^ *p* < 0.05; ^**^ *p* < 0.01; ^***^ *p* < 0.001. AAV8‐circDdb1‐oe: circDdb1 overexpression adeno‐associated virus 8; AAV8‐control: control AAV8.

We further investigated the pro‐atrophic role of circDdb1 in vivo. circDdb1 was overexpressed in gastrocnemius (GA) muscles by injecting the adeno‐associated virus 8 (AAV8) mediated circDdb1 in situ (**Figure** **3**A). We successfully increased circDdb1 expression via AAV8‐circDdb1 transduction, without affecting the mRNA level of linear Ddb1 in muscle (Figure 3B). Notably, overexpression of circDdb1 significantly reduced the wet muscle mass (Figure 3C). Then, we evaluated mice's muscle function and locomotion ability. Compared to the AAV8 control group, the circDdb1 overexpressing group's grip strength of the right hind leg was dramatically reduced (Figure 3D). Conducting endurance tests demonstrated that animals overexpressing circDdb1 could run noticeably less distance than mice overexpressing AAV8 control (Figure 3E). The tetanic forces were reduced in circDdb1 overexpression mice in the extensor digitorum longus (EDL) muscle (Figure 3F). Therefore, decreased muscle mass and function are the outcome of overexpressed circDdb1. We next examined muscle cryosections to confirm that circDdb1 overexpression significantly reduced myofiber cross‐sectional area (CSA) of myofiber as determined by wheat germ agglutinin (WGA) (Figure 3G). Furthermore, circDdb1 overexpression promoted the mRNA levels of Atrogin‐1 and MuRF‐1 (Figure 3H). We also explored the physiological processes involved in muscle protein degradation and synthesis during muscle atrophy. Interestingly, circDdb1 overexpression activated the UPS and ALP while suppressing the AKT/FOXO3A/mTOR signaling pathway, which is associated with protein synthesis (Figure 3I,J; Figure S4A–C, Supporting Information). Also, circDdb1 overexpression reduced the mtDNA content compared to the AAV8 control group (Figure 3K). Furthermore, circDdb1 overexpression reduced fiber type IIa size but had no effect on fiber‐type composition, according to immunofluorescence labeling with MHC (Figure S4D, Supporting Information). Altogether, our results suggest that circDdb1 is a functional pro‐atrophic factor in vivo.

### 2.3 Loss of circDdb1 Attenuates Muscle Atrophy

**Figure 4:**
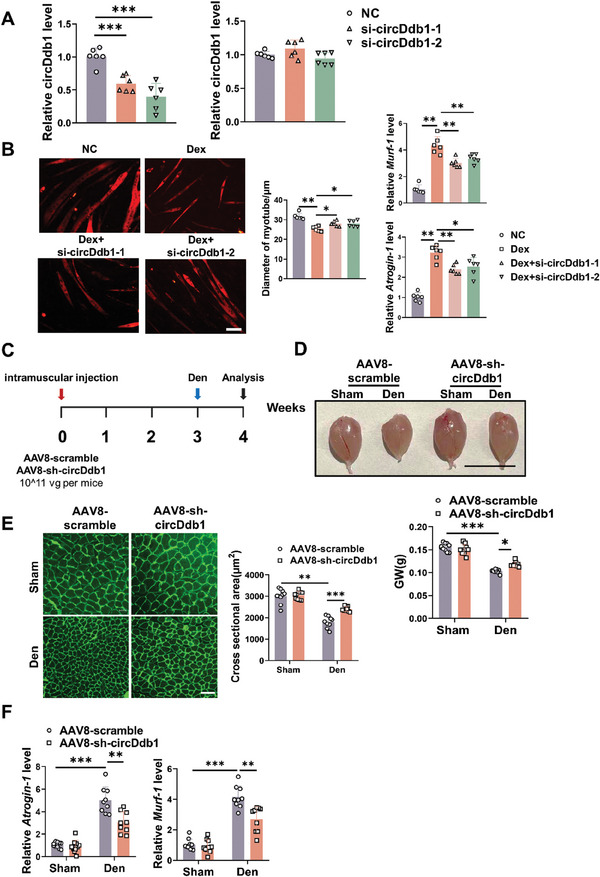
Downregulation of circDdb1 expression mitigates muscle atrophy both in vitro and in vivo. A) qRT‐PCR assessing circDdb1 and Ddb1 RNA expression levels in C2C12 myotubes transfected with si‐circDdb1 (*n* = 6). B) Representative images and statistical analysis of the diameter of C2C12 myotubes, and qRT‐PCR assessment of MuRF‐1 and Atrogin‐1 gene expression levels in C2C12 myotubes transfected with si‐circDdb1 in a model of dexamethasone (Dex)‐treated muscle atrophy (*n* = 6; scale bar: 100 µm). C) Schematic representation of the experimental design for viral injection and the construction of the model of muscle atrophy induced by denervation (Den). D) The gastrocnemius muscle morphology and weight (GW) of mice injected with AAV8‐sh‐circDdb1 in a model of denervation (Den)‐induced muscle atrophy (*n* = 10, 10, 9, 9; scale bar, 1 cm). E) Representative images and statistical analysis of myofiber cross‐sectional area of mice injected with AAV8‐sh‐circDdb1 in a model of denervation (Den)‐induced muscle atrophy (*n* = 10, 10, 9, 9; scale bar: 100 µm). F) qRT‐PCR assessing MuRF‐1 and Atrogin‐1 gene expression levels in gastrocnemius muscle of mice injected with AAV8‐sh‐circDdb1 in a model of denervation (Den)‐induced muscle atrophy (*n* = 10, 10, 9, 9). Statistical analysis was conducted employing a one‐way ANOVA test followed by the Bonferroni test for A and B, and a two‐way ANOVA test followed by the Tukey post hoc test involving C and D. Data are represented as mean ± SD. ^*^ *p* < 0.05; ^**^ *p* < 0.01; ^***^ *p* < 0.001. si‐circDdb1: small interfering RNA against circDdb1(including si‐circDdb1‐1 and si‐circDdb1‐2); NC: negative control small interfering RNA. AAV8‐sh‐circDdb1: circDdb1 knockdown adeno‐associated virus 8; AAV8‐scramble: control AAV8.

Next, we postulated that muscle atrophy may be mitigated by circDdb1 suppression. To test this hypothesis, we designed two specific siRNAs to knock down circDdb1 expression in C2C12 myotube cells. These two siRNAs can decrease circDdb1 but without affecting the linear Ddb1 mRNA (**Figure** **4**A). Notably, circDdb1 knockdown myotube cells exhibited protective effects against Dex, TNF‐α, and AngII stimulation, as evidenced by increased myotube diameter and decreased expression of Atrogin‐1 and MuRF‐1 (Figure 4B; Figure S5A,B, Supporting Information). These findings imply that downregulating circDdb1 may attenuate muscle atrophy in vitro. In addition, circDdb1 knockdown didn't affect the myoblast differentiation (Figure S5C, Supporting Information).

To extend our findings in vivo, AAV8‐mediated circDdb1 knockdown by shRNA was injected intramuscularly into GA muscle to reduce the expression of circDdb1 in mice. Terminal experiments were carried out one week following the denervation operation, following three weeks of AAV8 injection, and the denervation (Den) surgery (Figure 4C). When AAV8‐sh‐circDdb1 was administered to GA muscle during Den therapy, the expression of circDdb1 was markedly reduced (Figure S5D, Supporting Information). To reinforce the anti‐atrophy function of circDdb1, we found inhibition of circDdb1 increased muscle wet mass and myofiber CSA of the GA muscle under Den‐injury compared with scramble shRNA injection (Figure 4D,E). qRT‐PCR consistently revealed that GA muscles in Den‐treated mice treated with AAV8‐sh‐circDdb1 had substantially lower expression levels of both Atrogin‐1 and MuRF‐1 (Figure 4F). Specifically, the decreased phosphorylation of AKT (Ser‐473), FOXO3A (Ser‐253), mTOR, P70S6K, and 4EBP1 in Den‐induced muscle atrophy were also attenuated after knocking down circDdb1 (Figure S5E, Supporting Information). These findings point to a viable treatment for muscle atrophy caused by circDdb1 inhibition, as reducing circDdb1 may increase muscle weight in the Den‐induced muscle atrophy model.

In order to further confirm the protective effects in different atrophy models, we also performed the circDdb1 knockdown experiments with AAV8‐sh‐circDdb1 in immobilization (Imo) ‐induced muscle atrophy and angiotensin II (Ang II)‐induced muscle atrophy mice models. As in the above models, similar protective effects were observed (Figures S6–S8, Supporting Information). Notably, the grip strength, exercise ability, and tetanic force of mice were also improved in Ang II‐induced muscle atrophy after being injected with AAV8‐sh‐circDdb1 (Figure S7D–F, Supporting Information). Together, these results suggested that loss of circDdb1 attenuates muscle atrophy and improves muscle function.

### 2.4 CircDdb1 Encoded a Novel Peptide Named Circddb1‐867aa

**Figure 5:**
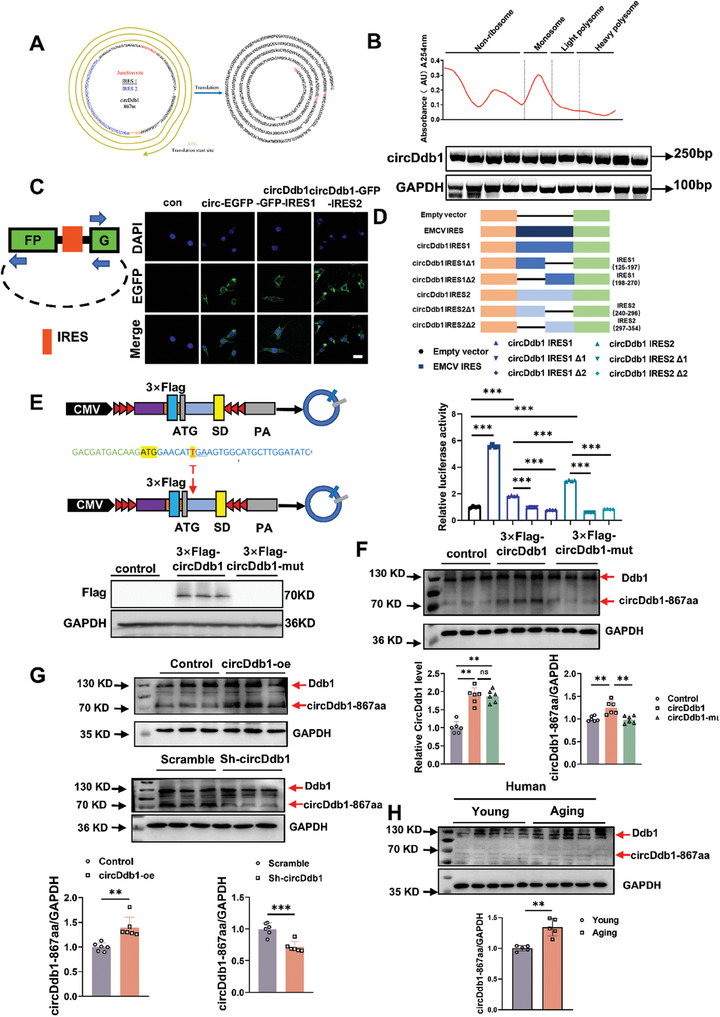
circDdb1 encodes a circDdb1‐867aa protein. A) Schematic diagram of IRES and open reading frames in circDdb1. B) The distribution of circDdb1 on ribosomes was detected by PCR. C) Immunofluorescence staining to detect GFP expression of C2C12 cell transfected with the TR‐circGFP plasmid and circDdb1‐circGFP‐IRES. D) Luc/Rluc activities of each transfected plasmid (*n* = 4). E) Schematic diagram of vectors (3×Flag‐circDdb1, 3×Flag‐circDdb1‐mut), and western blot assessment of Flag (*n* = 3) in C2C12 differentiated myotubes treated with 3×Flag‐circDdb1 and 3×Flag‐circDdb1‐mut plasmid. F) Western blot assessment of endogenous circDdb1‐867aa in C2C12 differentiated myotubes treated with Flag‐circDdb1 and Flag‐circDdb1‐mut plasmid (*n* = 6); qRT‐PCR assessing circDdb1 expression levels in C2C12 differentiated myotubes treated with Flag‐circDdb1 and Flag‐circDdb1‐mut plasmid (*n* = 6). G) Western blot assessment of endogenous circDdb1‐867aa in C2C12 differentiated myotubes treated with circDdb1 overexpression plasmid and sh‐circDdb1 plasmid (*n* = 6). H) Western blot assessment of endogenous circDdb1‐867aa in young and aged men and women muscle lysates (*n* = 5). Statistical analysis was performed using an unpaired, two‐tailed Student's *t*‐test to compare between two groups (D, F, G and H). ^**^ *p* < 0.01; ^***^ *p* < 0.001. Data are represented as mean ± SD.

First, binding proteins and miRNAs of circDdb1 were analyzed using pulldown assay and AGO2‐RNA immunoprecipitation (RIP) in C2C12 myotubes to provide insight into the molecular mechanism of circDdb1's regulation of muscle atrophy. However, we did not find the binding protein and the miRNA sponge potential of circDdb1 in myotube cells (Figure S9, Supporting Information). We then utilized the TransCirc (https://www.biosino.org/ transcirc/) and circRNADb (http://reprod.njmu.edu.cn/cgi‐bin/circrnadb/circRNADb.php) databases to analyze the sequence characteristics of circDdb1. The results revealed that circDdb1 contains an internal ribosome entry site (IRES: 240–354 sites and 125–270 sites) and an open reading frame (ORF), suggesting its potential protein‐encoding capacity. As indicated by bioinformatic prediction, an 867 amino acid protein could be translated by circDdb1 from nucleotides 190‐189 (total length: 2601nt) by coding for three turns, in a “rolling translation” mechanism (**Figure** **5**A).

To assess circDdb1's protein‐coding capacity, we performed a polysome profiling analysis using sucrose density gradient centrifugation. Notably, circDdb1 was detected in all polysome fractions (Figure 5B), indicating its association with translating ribosomes. Furthermore, we employed a dual luciferase reporter vector system and a circular vector‐based GFP reporter assay to confirm the IRES activity of circDdb1 (Figure 5C,D). We also found that the IRES activity of circDdb1 was enhanced in muscle atrophy (Figure S10A, Supporting Information).

To confirm whether circDdb1 could undergo “rolling translation” to produce an 867aa protein in vivo, we constructed a 3 × Flag‐circDdb1 vector (Flag‐circDdb1) by adding a 3 × Flag‐tag before the “ATG” start codon. We also constructed a Flag‐circDdb1‐mut as a negative control, which created a stop codon (TGA) by inserting a T at the 7th position after the ATG (Figure 5E). Western blot analysis demonstrated that the Flag‐circDdb1 vector could translate a Flag‐tag (≈70kDa), while the Flag‐circDdb1‐mut abolished Flag‐tag expression (Figure 5E). Additionally, we employed flag antibody immunoprecipitation (IP) in Flag‐circDdb1 overexpressed myotube cells, and a ≈70 kDa band was found with specificity. This band was then analyzed using a liquid chromatograph‐mass spectrometer (LC‐MS) (Figure S10B, Supporting Information). The LC‐MS/MS data demonstrated that the emerging protein's amino acid sequence exactly matched the expected one, indicating that circDdb1‐867aa was translated from circDdb1 (Figure S10B, Supporting Information).

To investigate the endogenous expression of the new protein, circDdb1‐867aa, we screened an antibody that targeted the putative circDdb1‐translated protein (Figure S11A, Supporting Information). Overexpression of the Flag‐circDdb1 vector, but not the Flag‐circDdb1‐mut vector, increased circDdb1‐867aa levels in C2C12 myotubes without affecting the expression of endogenous DDB1 protein and circDdb1 expression (Figure 5F). Consistently, knockdown of circDdb1 using sh‐circDdb1 dramatically decreased circDdb1‐867aa levels in C2C12 myotubes, while overexpression of circDdb1 significantly increased its expression (Figure 5G). Interestingly, we observed that the endogenous circDdb1‐867aa protein was upregulated in different muscle atrophy models, while endogenous DDB1 levels remained unchanged (Figure S11B, Supporting Information). The same result was also shown in aging human muscles (Figure 5H). Besides, circDdb1 knockdown experiments with AAV8‐sh‐circDdb1 in different mice models display that circDdb1 knockdown reduced the circDdb1‐867aa protein in muscle atrophy models (Figure S11C, Supporting Information).

Collectively, these findings demonstrate that circDdb1 undergoes IRES‐dependent “rolling translation” in muscle cells to produce a novel 867aa protein, circDdb1‐867aa.

### 2.5 circDdb1‐867aa Promotes Muscle Atrophy

**Figure 6:**
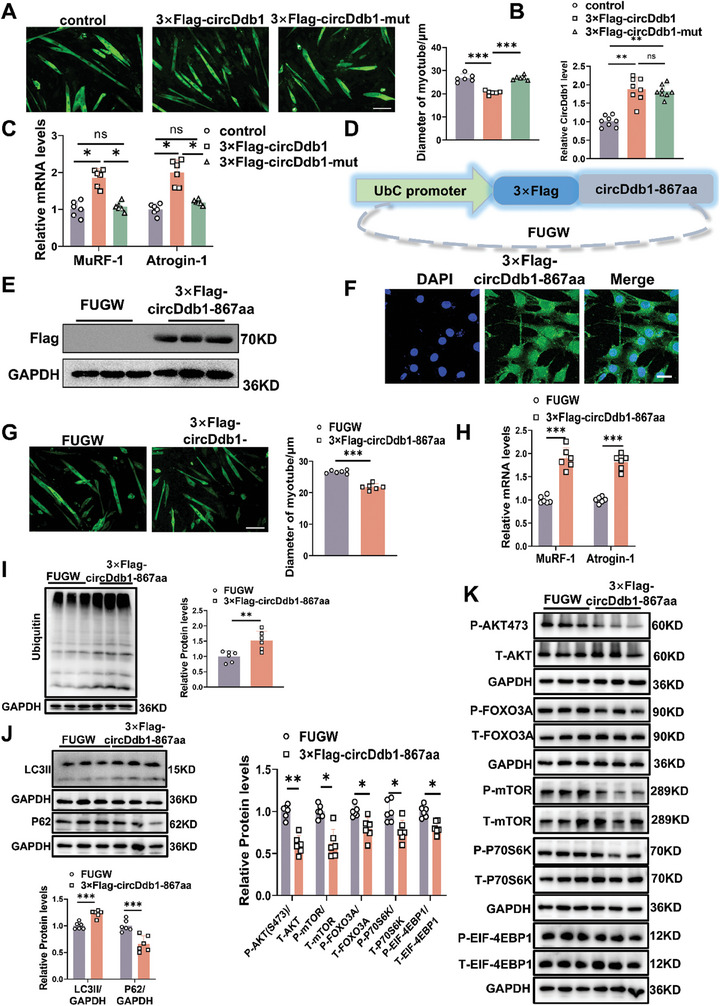
circDdb1‐867aa protein promotes muscle atrophy in vitro. A) Representative images and statistical analysis of the diameter of C2C12 myotubes in C2C12 myotubes transfected with 3×Flag‐circDdb1 and 3×Flag‐circDdb1‐mut plasmid. (*n* = 6; scale bar: 100 µm). B) qRT‐PCR assessing circDdb1 expression levels in C2C12 myotubes transfected with 3×Flag‐circDdb1 and 3×Flag‐circDdb1‐mut plasmid (*n* = 8). C) qRT‐PCR assessing MuRF‐1and Atrogin‐1 gene expression levels in C2C12 myotubes transfected with 3×Flag‐circDdb1 and 3×Flag‐circDdb1‐mut plasmid (*n* = 6). D) Fugw‐3×Flag‐circDdb1‐867aa plasmid. E) Western blot assessing circDdb1‐867aa content in C2C12 myotubes transfected with Fugw‐3×Flag‐circDdb1‐867aa lentivirus (*n* = 3). F) Representative images of the localization of circDdb1‐867aa in C2C12 cells (scale bar: 100 µm). G) Representative images and statistical analysis of the diameter of C2C12 myotubes in C2C12 myotubes transfected with Fugw‐3×Flag‐circDdb1‐867aa lentivirus (*n* = 4; scale bar: 100 µm). H) qRT‐PCR assessing MuRF‐1 and Atrogin‐1 gene expression levels in C2C12 myotubes transfected with Fugw‐3×Flag‐circDdb1‐867aa lentivirus (*n* = 6). I) Western blot assessing Ubiquitin protein expression level in C2C12 myotubes transfected with Fugw‐3×Flag‐circDdb1‐867aa lentivirus (*n* = 6). J) Western blot assessing P62 and LC3 protein expression levels in C2C12 myotubes transfected with Fugw‐3×Flag‐circDdb1‐867aa lentivirus (*n* = 6). K) Western blot assessing AKT/FOXO3A/mTOR pathway‐related protein expression levels (including p‐AKT, p‐FOXO3A, p‐mTOR, p‐P70S6K, p‐EIF‐4EBP1) in C2C12 myotubes transfected with Fugw‐3×Flag‐circDdb1‐867aa lentivirus (*n* = 6). Statistical analysis was conducted employing a one‐way ANOVA test followed by the Bonferroni test for (A–C), and an unpaired, two‐tailed Student's t‐test involving (G–K). Data are represented as mean ± SD. ^*^ *p* < 0.05; ^**^ *p* < 0.01; ^***^ *p* < 0.001.

To further explore the biological function of circDdb1‐867aa, the Flag‐circDdb1 and Flag‐circDdb1‐mut were transfected into fully differentiated C2C12 myotube cells. Based on myotube size and the degree of Atrogin‐1 and MuRF‐1 expression, the Flag‐circDdb1 vector could induce muscle atrophy in myotube cells, while the Flag‐circDdb1‐mut without translation function, lost the pro‐atrophy effects (**Figure** **6**A–C). These data suggested that circDdb1 could not promote muscle atrophy in myotube without its encoding protein circDdb1‐867aa.

In order to investigate the function of circDdb1‐867aa in more detail, we created a linear vector for the whole sequence of circDdb1‐867aa using 3 × Flag (Figure 6D). After transfecting 3×Flag‐circDdb1‐867aa into C2C12 myotube by lentivirus, we observed that 3 × Flag‐circDdb1‐867aa could overexpress circDdb1‐867aa in myotube cells and located in both nuclear and cytoplasm (Figure 6E,F; Figure S12A, Supporting Information). Furthermore, overexpression of circDdb1‐867aa could promote muscle atrophy in vitro, the results were indicated by decreasing the myotube diameter, upregulating the expression of Atrogin‐1, MuRF‐1, UPS‐ and ALP‐associated genes, inhibiting the AKT/FOXO3A/mTOR signaling pathway in myotube cells (Figure 6G–K; Figure S12B,C, Supporting Information).

**Figure 7:**
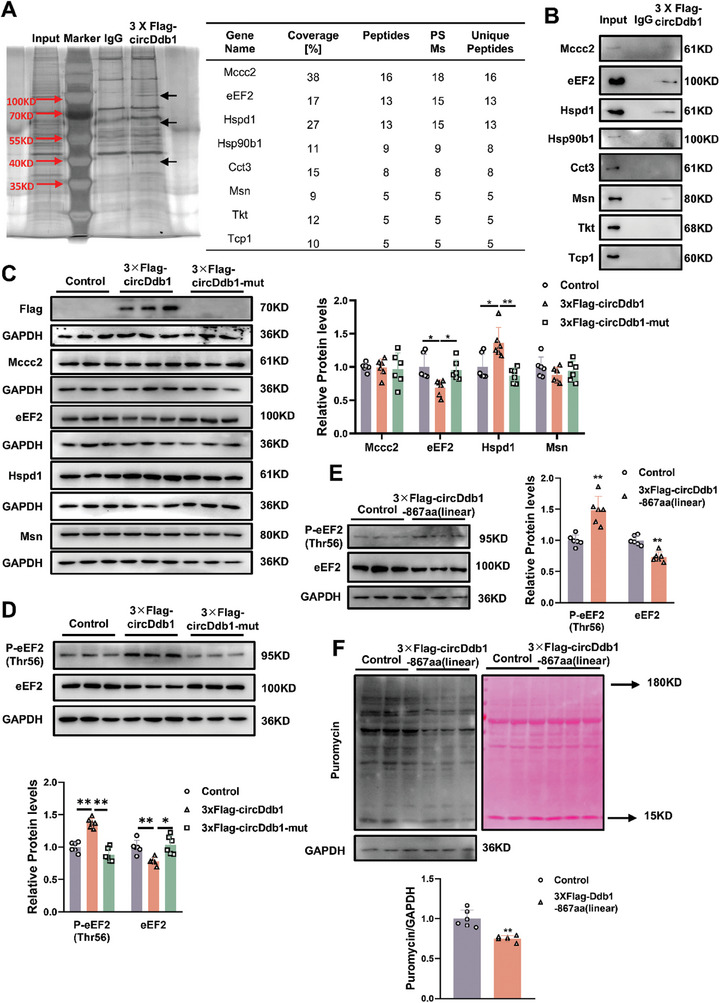
circDdb1‐867aa binds with eEF2. A) Silver staining the results of immunoprecipitated samples obtained with anti‐Flag antibodies. B) IP results in immunoprecipitated samples obtained with anti‐Flag antibodies. C) Western blot assessing Flag, Mccc2, eEF2, Hspd1 and Msn protein expression levels in C2C12 myotubes transfected with 3×Flag‐circDdb1 plasmid and 3×Flag‐circDdb1‐mut plasmid (*n* = 6). D) Western blot assessing P‐eEF2 (Thr56) and eEF2 protein expression levels in C2C12 myotubes transfected with 3×Flag‐circDdb1 plasmid and 3×Flag‐circDdb1‐mut plasmid (*n* = 6). E) Western blot assessing P‐eEF2 (Thr56) and eEF2 protein expression levels in C2C12 myotubes transfected with 3×Flag‐circDdb1‐867aa (linear) plasmid and control (*n* = 6). F) Western blot assessing puromycin expression level in C2C12 myotubes transfected with 3×Flag‐circDdb1‐867aa (linear) plasmid and control (*n* = 6). Statistical analysis was conducted employing an unpaired, two‐tailed Student's *t*‐test for B, E, and F, and a one‐way ANOVA test followed by Bonferroni test for C and D. Data are represented as mean ± SD. ^*^ *p* < 0.05; ^**^ *p* < 0.01.

To uncover the underlying mechanism of how circDdb1‐867aa regulates muscle atrophy, IP and LC‐MS were performed with flag antibodies in 3 × Flag‐circDdb1 overexpressed myotube cells (**Figure** **7**A). IP assay with flag antibody showed that Mcc2, eEF2, Hspd1, and Msn may be the interacting protein of circDdb1‐867aa (Figure 7B). In addition, we also found that after being treated with 3 × Flag‐circDdb1, eEF2 was decreased, and Hspd1 was increased, while this change was not observed when treated with 3 × Flag‐circDdb1‐Mut. This result suggested that eEF2 and Hspd1 were regulated by circDdb1‐867aa coding by circDdb1, not circDdb1 RNA only (Figure 7C). Additionally, all muscle atrophy models showed a drop in eEF2, while the majority of muscle atrophy models showed a decrease in Hspd1 (Figure S13A,B, Supporting Information). Translation elongation is regulated by eEF2, a GTPase that moves the peptidyl‐tRNA from the ribosome's A‐site to the P‐site. Different phosphorylation sites are targeted by numerous kinases that either favorably or negatively affect eEF2 activity. When eEF2 kinase (eEF2K) phosphorylates eEF2 at Thr56, eEF2 cannot bind a ribosome and stays inactive. We found that the phosphorylation level of eEF2 at Thr56 was increased in circDdb1 coding circDdb1‐867aa, not in mutant circDdb1 (Figure 7D). In addition, the phosphorylation level of eEF2 at Thr56 was increased in Den‐induced muscle atrophy models (Figure S13C, Supporting Information). Furthermore, circDdb1‐867aa overexpression promoted the phosphorylation level of eEF2 at Thr56 and hindered the translation level (Figure 7E,F). Together, these data suggested that circDdb1‐867aa binds with eEF2, and increases the phosphorylation level of eEF2 at Thr56, thus hindering translation and promoting muscle atrophy.

### 2.6 EIF4A3 Induced circDdb1 Expression in Muscle Atrophy

**Figure 8:**
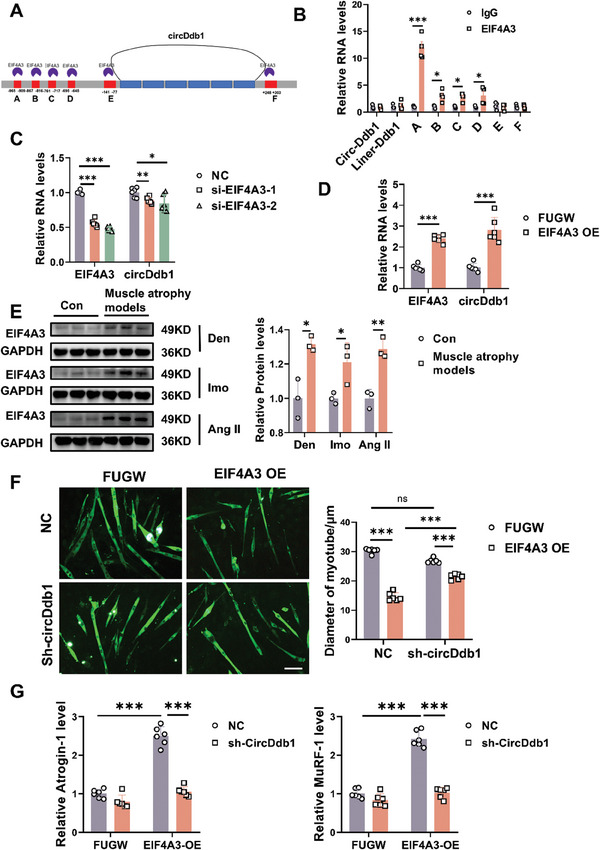
EIF4A3 induces circDdb1 expression in muscle atrophy. A) The binding sites for EIF4A3 in the flanking sequences of the DDB1 mRNA transcript were predicted using CircInteractome. B) RIP assay to verify EIF4A3 binding at the putative sites (*n* = 4). C) qRT‐PCR assessing EIF4A3 mRNA and circDdb1 expression levels in C2C12 myotubes transfected with si‐EIF4A3 and NC (*n* = 6). D) qRT‐PCR assessing EIF4A3 mRNA and circDdb1 expression levels in C2C12 myotubes transfected with EIF4A3 OE plasmid and Fugw (*n* = 6). E) Western blot assessing EIF4A3 protein expression level in animal muscle atrophy models (including Den‐, Imo‐, and AngII‐induced muscle atrophy models) (*n* = 6). F) Representative images and statistical analysis of the diameter of C2C12 myotubes in C2C12 myotubes transfected with EIF4A3 OE plasmid and sh‐circDdb1 plasmid (*n* = 6; scale bar: 100 µm). G) qRT‐PCR assessing Atrogin‐1 and MuRF‐1gene expression levels in C2C12 myotubes transfected with EIF4A3 OE plasmid and sh‐circDdb1 plasmid (*n* = 6). Statistical analysis was conducted employing an unpaired, two‐tailed Student's *t*‐test for B, D, and E, and a one‐way ANOVA test followed by Bonferroni test for C, and a two‐way ANOVA test followed by Tukey post hoc test for F and G. Data are represented as mean ± SD. ^*^ *p* < 0.05; ^**^ *p* < 0.01; ^***^ *p* < 0.001. si‐EIF4A3: small interfering RNA against EIF4A3 (including si‐EIF4A3‐1 and si‐EIF4A3‐2); NC: negative control small interfering RNA. EIF4A3 OE: EIF4A3 overexpression plasmid; Fugw: control vector. sh‐Ddb1: shRNA against Ddb1; NC: control shRNA.

The flank intron sequences of circRNAs can interact with RNA‐binding proteins, which can then affect the generation of circRNAs.^[^ ^16^ ^]^ Consequently, we searched the circInteractome (https://circinteractome.nia.nih.gov/index.html) and circAltas databases (http://159.226.67.237:8080/new/index.php) to identify putative RBP sites in the flanking regions of circDdb1 in order to investigate how circDdb1 was elevated in muscle atrophy. We found that 6 binding sites and 1 binding site for EIF4A3 are present in the upstream region and downstream of the circDdb1 mRNA transcript, respectively (**Figure** **8**A; Figure S14A,B, Supporting Information). The RIP experiment demonstrated that EIF4A3 could attach to mRNA through the binding area A–D, whereas regions E, F, and circDdb1 did not exhibit any interaction (Figure 8B). RNA‐protein pulldown assay and Electrophoretic mobility shift (EMSA) assay further verified the binding between EIF4A3 and area A–D (Figure S14C,D, Supporting Information). These results indicate that EIF4A3 can bind to the flanking sequences of circDdb1. Furthermore, EIF4A3 didn't affect the nuclear export of circDdb1(Figure S14E, Supporting Information).

In addition, we further found that overexpression of EIF4A3 promoted circDdb1, while inhibition of EIF4A3 reduced the circDdb1expression in C2C12 myotube cells (Figure 8C,D). In Parallel, the EIF4A3 expression level was increased in muscle atrophy models (Figure 8E). To further clarify the functional interaction between EIF4A3 and circDdb1, we performed the rescue experiment by intervening the EIF4A3 and circDdb1 independently or respectively. It was found that overexpression of EIF4A3 could induce muscle atrophy while inhibiting circDdb1 abolished the pro‐atrophy effects, induced by EIF4A3 upregulation (Figure 8F,G). Similarly, repression of EIF4A3 could attenuate muscle atrophy in vitro and circDdb1 overexpression could abolish the protective effect of EIF4A3 inhibition in muscle atrophy (Figure S15, Supporting Information). These data suggest that EIF4A3 promotes muscle atrophy via promoting circDdb1. In conclusion, EIF4A3 induced circDdb1 expression in muscle atrophy via binding to the flanking sequences of circDdb1.

## 3 Discussion

In the present study, we unraveled the novel functional roles of a circular RNA, circDdb1, in regulating muscle atrophy. We found that RNA binding protein EIF4A3 promoted circDdb1 production, which encoded a novel protein termed circDdb1‐867aa. Thereafter circDdb1‐867aa bound with eEF2, which increased the phosphorylation level of eEF2 at Thr56 to decrease protein translation and promote muscle atrophy.

Prior studies showed that some circRNAs could regulate muscle growth and development by different regulatory modes. For example CircZfp609,^[^ ^17^ ^]^ CircLMO7,^[^ ^18^ ^]^ CircFGFR4,^[^ ^19^ ^]^ CircFUT10,^[^ ^20^ ^]^ CircSVIL,^[^ ^21^ ^]^ CircRBFOX2,^[^ ^22^ ^]^ and CircFGFR2^[^ ^23^ ^]^ regulate myogenesis by sponging miRNAs. CircSamd4 and circMYBPC promote differentiation by interacting with PURA/PURB and MyHC, separately.^[^ ^24^ ^]^ Circ‐ZNF609 and Circ‐FAM188B stimulate myogenesis by encoding novel polypeptides.^[^ ^25^ ^]^ These studies suggested that circRNAs could maintain muscle homeostasis and mediate muscle repair. In muscle disease, circRNA expression profiles in Duchenne muscular dystrophy (DMD) were conducted. A total of 197 circRNAs with differential expression (FC > 1.5 and *p*‐value < 0.05) were found.^[^ ^26^ ^]^ Reassaying ribs‐depleted libraries from 25 Myotonic Dystrophy Type 1 tibialis anterior biopsies and five controls using RNA sequencing (RNAseq) data revealed four circRNAs (CDYL, HIPK3, RTN4_03, and ZNF609) that were elevated in DM1 muscles.^[^ ^27^ ^]^ In addition, a DMD circular RNA was found to be generated from the exon‐45–55 of the dystrophin (DMD) gene.^[^ ^28^ ^]^ Despite the rapidly increasing number of circRNAs found in skeletal muscle, research on circRNAs in muscle atrophy is still in its infancy with a handful being characterized to date. CircTmeff1, another circRNA we found before, was the first circRNA that upregulated in multiple types of muscle atrophy.^[^ ^15^ ^]^ In the current study, using circRNA seq from denervation‐induced muscle atrophy, and qRT‐PCR from multiple types of muscle atrophy, we identified a new circular RNA, circDdb1, that promoted muscle atrophy in vitro and in vivo. circDdb1 could therefore represent a new treatment for muscle atrophy and should be studied further.

Mammals possess circRNAs in large quantities, which function in many ways to control transcriptional and post‐transcriptional levels of gene regulation. To investigate the molecular mechanisms of circDdb1 in inducing muscle atrophy, pulldown assay and AGO2‐RNA immunoprecipitation (RIP) were performed and no binding protein or sponge potential of circDdb1 was identified in myotube cells. We then focused on the encoding capability of circDdb1. The translation of circRNA is a cap‐independent translation initiation mode, which is triggered by the Internal Ribosome Entry site (IRES) or N6‐methyladenosines (m6A).^[^ ^29^ ^]^ To date, in skeletal muscle, some novel proteins that are translated by circRNAs have been uncovered.^[^ ^30^ ^]^ Circ‐ZNF609 and CircFAM188B could translate into proteins that promote myogenesis via an IRES‐dependent translation module.^[^ ^25^ ^]^ Although the translation via cap‐independent initiation is less efficient than the cap‐dependent one, this module expands the content of the translatome. Ribosome profiling/ribosome nascent‐chain complex‐bound RNA sequencing (RNC‐seq) and circRNA seq were introduced to uncover the potential coding of circRNAs.^[^ ^31^ ^]^ In the current work, we found that the “rolling‐translation” module could translate circDdb1 into circDdb1‐867aa in an IRES‐dependent way. It is reported that covalently closed circular (CCC) RNAs from rice yellow mottle virus (RYMV) have an unusual ORF with the sequence UGAUGA. This unique ORF was capable of initiating translation from the AUG in the sequence UGAUGA of the 220‐nt circular RNA by the internal ribosome binding site (IRBS). Given that the 220‐nt sequence is not a multiple of three, subsequent translation rounds could potentially realign the reading frame.^[^ ^32^ ^]^ To be noticed, when the nucleotide sequence of the circRNA is not an exact multiple of three, the rolling cycle translation mechanism allows for a maximum of three rounds of translation (+0, +1, and +2 frame). In the context of eukaryotic cells, a similar phenomenon has been observed where circ‐EGFR encodes a polymetric protein complex known as rolling‐translated EGFR. This process involves ORF rolling translation and a programmed‐1 ribosomal frameshifting mechanism that induces an out‐of‐frame stop codon.^[^ ^33^ ^]^ Our finding resonates with prior studies that circRNA undergoes the complexity and versatility of translation mechanisms in different biological systems. Importantly, we further found that circDdb1‐867aa was a key functional mediator of circDdb1 in muscle atrophy, and inhibition of this novel protein derived from circDdb1 eliminated atrophy in myotube cells. In addition, we further found that loss of the protein‐coding function of circDdb1 eliminated atrophy in myotube cells. This suggests that the nascent protein, circDdb1‐867aa, is a key mediator of circDdb1 in muscle atrophy.

Thereafter circDdb1‐867aa bound with eEF2, which increased the phosphorylation level of eEF2 at Thr56 to decrease protein translation and promote muscle atrophy. In addition, we also observed that Hspd1 could interact with the circDdb1‐867aa. Hsdp1, also called heat shock protein 60 (Hsp60), is considered one of the mitochondria's chaperone molecules. As a molecular chaperone, Hsdp1 could participate in the maintenance of protein homeostasis, which forms large oligomers in the other cell compartments and functions together with Hsp10 in protein folding. Interestingly, we observed that increased protein level of Hspd1 in 3×Flag‐circDdb1 treatment, not in 3×Flag‐circDdb1‐Mut in Figure 7C. Thus, we hypothesized that Hspd1 may help the protein folding of circDdb1‐867aa. When exogenous expression of circDdb1‐867aa was increased, the Hsdp1 was recruited and participated in protein folding. Notably, the muscle diseases are also correlating with Hspd1.^[^ ^34^ ^]^ Hsdp1 was decreased in the diaphragm muscle of Dystrophic‐trained mice and in the extensor digitorum longus muscle of Diabetic rats.^[^ ^35^ ^]^ These results seem to be consistent with our findings that Hspd1 expression was inhibited in our muti‐type of muscle atrophy models (Figure S13B). Regarding our data, we assume that circDdb1‐867‐induced muscle atrophy is not mediated by Hsdp1, but the putative interaction between circDdb1‐867 and Hsdp1 needs to be further investigated.

RNA‐binding proteins, including protein quaking (HQK; encoded by QKI) or RNA‐binding protein FUS, have the ability to control the production of circular RNA.^[^ ^36^ ^]^ Eukaryotic initiation factor 4A‐3 (EIF4A3) is an RBP that is mostly found in the nucleus. It is also a fundamental part of the exon junction complex (EJC), which is engaged in the nuclear export of spliced mRNA and mRNA splicing.^[^ ^37^ ^]^ Interestingly, EIF4A3 was found to promote the circRNA production, such as circ_0084615,^[^ ^38^ ^]^ circARHGAP29,^[^ ^39^ ^]^ circular RNA ASAP1,^[^ ^40^ ^]^ circMMP9,^[^ ^41^ ^]^ circular RNA PRKAR1B.^[^ ^42^ ^]^ In our study, we discovered that EIF4A3 might interact with circDdb1's upstream flanking regions to control the expression of that gene. In addition, EIF4A3 aggregates in the muscle cells might contribute to facioscapulohumeral dystrophy (FSHD) pathophysiology.^[^ ^43^ ^]^ In accordance with the muscle disease, we also found that EIF4A3 was aggregated in muscle atrophy models. Furthermore, overexpression of EIF4A3 could promote muscle atrophy. Our rescue experiments showed that EIF4A3 promoted muscle atrophy by promoting circDdb1 expression. Therefore, we concluded that EIF4A3 induced circDdb1 formation in muscle atrophy. However, the exact mechanism of how EIF4A3 regulates the circularization of circDdb1 transcript is still unknown and requires further mechanistic studies.

In conclusion, we found that muscle atrophy was characterized by high expression of circDdb1, whereas inhibition of circDdb1 could serve as a potential therapeutic method for preventing multiple types of muscle atrophy.

## 4 Experimental Section

### Animal Models

The NIH's guidelines for the use and care of laboratory animals in biomedical research (no. 85‐23, revised 1996) served as the basis for all animal‐related procedures, and Shanghai University's ethical committees approved the experimental protocol (approval number: ECSHU2020‐100). Eight‐week‐old male C57BL/6 mice were acquired from Charles River in Beijing, China, and housed at Shanghai University's SPF experimental animal facility (Shanghai, China). The muscle atrophy models were established according to the previous work.^[^ ^15^, ^44^ ^]^ Briefly, mice were anesthetized with isoflurane (3–4% isoflurane for induction, 1–2% isoflurane for maintenance). The denervation‐induced muscle atrophy was established by cutting the sciatic nerve of mice. The same procedure was used to create sham mice, but this time the sciatic nerve was left intact. The hind limbs of mice were secured at 90° of flexion for the immobilization‐induced muscle atrophy paradigm by screwing a 0.4 × 8 mm screw through the talus and calcaneus into the tibia's shaft. Sham animals were not given this treatment. In the Angiotensin II‐induced muscle atrophy paradigm, an osmotic minipump (ALZET, #2001) was implanted into the mice to infuse them with Angiotensin II (Angiotensin II human Acetate, Selleck, #P1085 200 µL, 2 mg mL^−1^) at a rate of 1.46 mg kg^−1^ day^−1^ for a week. PBS‐buffered saline (PBS) was used for osmotic minipump perfusion in the control mice. The mice were sacrificed 1 week after these models. Mice were sacrificed via intraperitoneal sodium pentobarbital (60 mg kg^−1^) for muscle tissue collection. The right hindlimb's gastrocnemius was removed, and the body and muscle weights were recorded. Muscle samples were either embedded for histological examinations or instantly snap‐frozen in liquid nitrogen and kept at −80 °C for further analysis.

### Human Samples

Skeletal muscle tissue near the knee joint was collected with the use of arthroscopy during an operation in the Shanghai Gongli Hospital. The subjects were divided into young and aging groups based on age. All procedures conformed to the 1964 Helsinki Declaration and its later amendments or comparable ethical standards and were approved by the Ethics Committee of Shanghai Gongli Hospital (GLYYls2024‐016).

### Statistical Analysis

Data are represented as mean ± SD. All analyses were performed using GraphPad Prism 8.0. An unpaired, two‐tailed Student's *t*‐test was used for comparison between the two groups. Two‐way ANOVA with the Tukey test was performed to compare multiple groups. Differences with *p* <0.05 were considered significant.

### Study Approval

All animal experiments were approved by Shanghai University's ethical committees approved the experimental protocol (approval number: ECSHU2020‐100). Human samples were approved by the Ethics Committee of Shanghai Gongli Hospital (GLYYls2024‐016), and all participants provided written informed consent.

Detailed experimental methods are available in Supporting Information.

## Conflict of Interest

The authors declare no conflict of interest.

## Author contributions

X. Z., T. Y., and Y.Z. contributed equally to this work. J.X. and J.L. conceptualized the study, designed the research, and wrote the manuscript. J.L., X.Z., T.Y., Y.Z., Q.N., J.C., Q.L., X.R., X.Y., S.W., Y.Y., and Z.L. performed experiments and analyzed the data. M.W. collected the human samples. D.L., Y.Y., L.C., E.C., G.L., D.C., and T.S.B. revised the manuscript.

## Supporting information



Supporting Information

## Data Availability

The data that support the findings of this study are available from the corresponding author upon reasonable request.

## References

[1] S. Schiaffino , K. A. Dyar , S. Ciciliot , B. Blaauw , M. Sandri , FEBS J. 2013, 280, 4294. 23517348 10.1111/febs.12253

[2] a) K. Yuasa , K. Okubo , M. Yoda , K. Otsu , Y. Ishii , M. Nakamura , Y. Itoh , K. Horiuchi , Sci. Rep. 2018, 8, 9037; 29899565 10.1038/s41598-018-26632-w PMC5998077

[3] a) P. Bonaldo , M. Sandri , Dis. Model Mech. 2013, 6, 25, ; 23268536 10.1242/dmm.010389 PMC3529336

[4] L. C. Hunt , B. Schadeberg , J. Stover , B. Haugen , V. Pagala , Y. D. Wang , J. Puglise , E. R. Barton , J. Peng , F. Demontis , Nat. Commun. 2021, 12, 1418. 33658508 10.1038/s41467-021-21738-8 PMC7930053

[5] L. Chen , L. Chen , L. Wan , Y. Huo , J. Huang , J. Li , J. Lu , B. Xin , Q. Yang , C. Guo , Oncol Rep 2019, 42, 479. 31233199 10.3892/or.2019.7205 PMC6610044

[6] a) S. C. Bodine , T. N. Stitt , M. Gonzalez , W. O. Kline , G. L. Stover , R. Bauerlein , E. Zlotchenko , A. Scrimgeour , J. C. Lawrence , D. J. Glass , G. D. Yancopoulos , Nat. Cell Biol. 2001, 3, 1014; 11715023 10.1038/ncb1101-1014

[7] a) M. Zhang , Y. Xu , Y. Zhang , B. Li , G. Lou , Cell. Signal. 2021, 84, 110014; 33894314 10.1016/j.cellsig.2021.110014

[8] a) S. Aufiero , Y. J. Reckman , Y. M. Pinto , E. E. Creemers , Nat. Rev. Cardiol. 2019, 16, 503; 30952956 10.1038/s41569-019-0185-2

[9] S. Peng , C. Song , H. Li , X. Cao , Y. Ma , X. Wang , Y. Huang , X. Lan , C. Lei , B. Chaogetu , Molecular Therapy‐Nucleic Acids 2019, 16, 481. 31051333 10.1016/j.omtn.2019.03.009 PMC6495097

[10] B. Chen , J. Yu , L. Guo , M. S. Byers , Z. Wang , X. Chen , H. Xu , Q. Nie , Cells 2019, 8, 177. 30791438 10.3390/cells8020177 PMC6406597

[11] X. Shen , X. Zhang , W. Ru , Y. Huang , X. Lan , C. Lei , H. Chen , Molecular Therapy‐Nucleic Acids 2020, 19, 986. 32036250 10.1016/j.omtn.2019.12.032 PMC7013137

[12] X. Chen , H. Ouyang , Z. Wang , B. Chen , Q. Nie , Cells 2018, 7, 199. 30404220 10.3390/cells7110199 PMC6262629

[13] Z. Zhang , Y. Fan , K. Deng , Y. Liang , G. Zhang , X. Gao , M. El‐Samahy , Y. Zhang , M. Deng , F. Wang , FASEB J. 2022, 36, e22097. 34935184 10.1096/fj.202101317R

[14] I. Legnini , G. Di Timoteo , F. Rossi , M. Morlando , F. Briganti , O. Sthandier , A. Fatica , T. Santini , A. Andronache , M. Wade , Mol. Cell 2017, 66, 22. 28344082 10.1016/j.molcel.2017.02.017 PMC5387670

[15] R. Chen , T. Yang , B. Jin , W. Xu , Y. Yan , N. Wood , H. I. Lehmann , S. Wang , X. Zhu , W. Yuan , H. Chen , Z. Liu , G. Li , T. S. Bowen , J. Li , J. Xiao , Adv. Sci. (Weinh) 2023, e2206732. 37088818 10.1002/advs.202206732 PMC10265041

[16] L. Yang , J. E. Wilusz , L. L. Chen , Annu. Rev. Cell Dev. Biol. 2022, 38, 263. 35609906 10.1146/annurev-cellbio-120420-125117 PMC10119891

[17] Y. H. Wang , M. L. Li , Y. H. Wang , J. Liu , M. L. Zhang , X. T. Fang , H. Chen , C. L. Zhang , Int. J. Biol. Macromol. 2019, 121, 1308. 30201567 10.1016/j.ijbiomac.2018.09.039

[18] X. F. Wei , H. Li , J. M. Yang , D. Hao , D. Dong , Y. Z. Huang , X. Y. Lan , M. Plath , C. Z. Lei , F. P. Lin , Y. Y. Bai , H. Chen , Cell Death Dis. 2017, 8, 10.1038/cddis.2017.541. PMC5680912 29072698

[19] H. Li , X. F. Wei , J. M. Yang , D. Dong , D. Hao , Y. Z. Huang , X. Y. Lan , M. Plath , C. Z. Lei , Y. Ma , F. P. Lin , Y. Y. Bai , H. Chen , Molecular Therapy‐Nucleic Acids 2018, 11, 272. 29858062 10.1016/j.omtn.2018.02.012 PMC5992882

[20] H. Li , J. M. Yang , X. F. Wei , C. C. Song , D. Dong , Y. Z. Huang , X. Y. Lan , M. Plath , C. Z. Lei , Y. Ma , X. L. Qi , Y. Y. Bai , H. Chen , J. Cell. Physiol. 2018, 233, 4643. 29044517 10.1002/jcp.26230

[21] H. J. Ouyang , X. L. Chen , W. M. Li , Z. H. Li , Q. H. Nie , X. Q. Zhang , Front. Genet. 2018, 9, 10.3389/fgene.2018.00172. PMC5964199 29868120

[22] H. J. Ouyang , X. L. Chen , Z. J. Wang , J. Yu , X. Z. Jia , Z. H. Li , W. Luo , B. A. Abdalla , E. Jebessa , Q. H. Nie , X. Q. Zhang , DNA Res. 2018, 25, 71. 29036326 10.1093/dnares/dsx039 PMC5824844

[23] X. L. Chen , H. J. Ouyang , Z. J. Wang , B. A. Chen , Q. H. Nie , Cells 2018, 7, 199.30404220

[24] a) P. R. Pandey , J. H. Yang , D. Tsitsipatis , A. C. Panda , J. H. Noh , K. M. Kim , R. Munk , T. Nicholson , D. Hanniford , D. Argibay , X. L. Yang , J. L. Martindale , M. W. Chang , S. W. Jones , E. Hernando , P. Sen , S. De , K. Abdelmohsen , M. Gorospe , Nucleic Acids Res. 2020, 48, 3789; 31980816 10.1093/nar/gkaa035 PMC7144931

[25] a) I. Legnini , G. Di Timoteo , F. Rossi , M. Morlando , F. Briganti , O. Sthandier , A. Fatica , T. Santini , A. Andronache , M. Wade , P. Laneve , N. Rajewsky , I. Bozzoni , Mol. Cell 2017, 66, 22; 28344082 10.1016/j.molcel.2017.02.017 PMC5387670

[26] Z. B. Song , Y. M. Liu , X. B. Fang , M. S. Xie , Z. Y. Ma , Z. G. Zhong , X. L. Feng , W. X. Zhang , Funct. Integr. Genomics 2020, 20, 397. 31736012 10.1007/s10142-019-00724-w

[27] C. Voellenkle , A. Perfetti , M. Carrara , P. Fuschi , L. V. Renna , M. Longo , S. B. Sain , R. Cardani , R. Valaperta , G. Silvestri , I. Legnini , I. Bozzoni , D. Furling , C. Gaetano , G. Falcone , G. Meola , F. Martelli , Int. J. Mol. Sci. 2019, 20, 1938. 31010208 10.3390/ijms20081938 PMC6515344

[28] H. Suzuki , Y. Aoki , T. Kameyama , T. Saito , S. Masuda , J. Tanihata , T. Nagata , A. Mayeda , S. Takeda , T. Tsukahara , Int. J. Mol. Sci. 2016, 17, 1722. 27754374 10.3390/ijms17101722 PMC5085753

[29] a) M. Lei , G. T. Zheng , Q. Q. Ning , J. N. Zheng , D. Dong , Molecular Cancer 2020, 19, 10.1186/s12943-020-1135-7; PMC7023758 32059672

[30] X. M. Sun , Y. Kang , M. X. Li , Y. J. Li , J. Z. Song , Bba‐Gene Regul Mech 2022, 1865, 10.1016/j.bbagrm.2022.194888. 36280131

[31] X. Y. Gao , X. Xia , F. Y. Li , M. L. Zhang , H. K. Zhou , X. J. Wu , J. Zhong , Z. Zhao , K. Zhao , D. W. Liu , F. Z. Xiao , Q. Xu , T. Jiang , B. Li , S. Y. Cheng , N. Zhang , Nat. Cell Biol. 2021, 23, 278. 33664496 10.1038/s41556-021-00639-4

[32] M. G. AbouHaidar , S. Venkataraman , A. Golshani , B. Liu , T. Ahmad , Proc Natl Acad Sci 2014, 111, 14542. 25253891 10.1073/pnas.1402814111 PMC4209996

[33] Y. Liu , Z. Li , M. Zhang , H. Zhou , X. Wu , J. Zhong , F. Xiao , N. Huang , X. Yang , R. Zeng , L. Yang , Z. Xia , N. Zhang , Neuro Oncol 2021, 23, 743. 33325513 10.1093/neuonc/noaa279 PMC8099477

[34] A. Marino Gammazza , F. Macaluso , V. Di Felice , F. Cappello , R. Barone , Cells 2018, 7, 224. 30469470 10.3390/cells7120224 PMC6315887

[35] a) G. Morici , M. Frinchi , A. Pitruzzella , V. Di Liberto , R. Barone , A. Pace , V. Di Felice , N. Belluardo , F. Cappello , G. Mudo , M. R. Bonsignore , J. Cell. Physiol. 2017, 232, 2044; 27576008 10.1002/jcp.25573

[36] a) C. X. Liu , L. L. Chen , Cell 2022, 185, 2016; 35584701 10.1016/j.cell.2022.04.021

[37] a) Y. Lin , R. Liang , Y. W. Mao , J. Z. Ye , R. Y. Mai , X. Gao , Z. Y. Liu , T. Wainwright , Q. Li , M. Luo , L. Y. Ge , Y. Q. Li , D. H. Zou , J. Cell. Biochem. 2020, 121, 4094; 31898336 10.1002/jcb.29596

[38] Z. P. Jiang , Q. W. Tai , X. J. Xie , Z. H. Hou , W. Liu , Z. M. Yu , Z. Q. Liang , S. Chen , Journal of Experimental & Clinical Cancer Research 2021, 40, 10.1186/s13046-021-02029-y. PMC8273970 34253241

[39] X. K. Jiang , S. Q. Guo , S. Wang , Y. Y. Zhang , H. J. Chen , Y. Wang , R. L. Liu , Y. J. Niu , Y. Xu , Cancer Res. 2022, 82, 831. 34965937 10.1158/0008-5472.CAN-21-2988

[40] Y. T. Wei , C. F. Lu , P. Zhou , L. Zhao , X. Lyu , J. X. Yin , Z. M. Shi , Y. P. You , Neuro‐Oncology 2021, 23, 611. 32926734 10.1093/neuonc/noaa214 PMC8041353

[41] R. J. Wang , S. Zhang , X. Y. Chen , N. Li , J. W. Li , R. C. Jia , Y. Q. Pan , H. Q. Liang , Molecular Cancer 2018, 17, 10.1186/s12943-018-0911-0. PMC6260852 30470262

[42] Z. H. Feng , L. Zheng , T. Yao , S. Y. Tao , X. A. Wei , Z. Y. Zheng , B. J. Zheng , X. Y. Zhang , B. Huang , J. H. Liu , Y. L. Chen , Z. Shan , P. T. Yuan , C. G. Wang , J. Chen , S. Y. Shen , F. D. Zhao , Cell Death Dis. 2021, 12, 10.1038/s41419-021-04339-7.

[43] S. C. Shadle , J. W. Zhong , A. E. Campbell , M. L. Conerly , S. Jagannathan , C. J. Wong , T. D. Morello , S. M. van der Maarel , S. J. Tapscott , PLoS Genet. 2017, 13, e1006658. 28273136 10.1371/journal.pgen.1006658 PMC5362247

[44] a) J. Li , L. Wang , X. Hua , H. Tang , R. Chen , T. Yang , S. Das , J. Xiao , Mol. Ther. 2020, 28, 1359; 32222157 10.1016/j.ymthe.2020.03.005 PMC7210721

