# Supplementary material for: EIF4A3‐Induced Circular RNA CircDdb1 Promotes Muscle Atrophy through Encoding a Novel Protein CircDdb1‐867aa
Source: Adv Sci (Weinh). 2024 Oct 16;11(45):2406986. doi: 10.1002/advs.202406986 (PMC11615752; doi:10.1002/advs.202406986)
Supplement: Supplementary file 1 — Supporting Information [file ADVS-11-2406986-s001.pdf]

ADVANCED SCIENCE
Open Access

Supporting Information

for *Adv. Sci.*, DOI 10.1002/advs.202406986

EIF4A3-Induced Circular RNA CircDdb1 Promotes Muscle Atrophy through Encoding a Novel Protein CircDdb1-867aa

Xiaolan Zhu, Tingting Yang, Yongjun Zheng, Qiumeng Nie, Jingying Chen, Qian Li, Xinyi Ren, Xiaohang Yin, Siqi Wang, Yuwei Yan, Zhengyu Liu, Ming Wu, Dongchao Lu, Yan Yu, Lei Chen, Emeli Chatterjee, Guoping Li, Dragos Cretoiu, T Scott Bowen, Jin Li and Junjie Xiao**

Supporting Information

EIF4A3-induced circular RNA circDdb1 promotes muscle atrophy through encoding a novel protein circDdb1-867aa

Xiaolan Zhu, Tingting Yang, Yongjun Zheng, Qiumeng Nie, Jingying Chen, Qian Li, Xinyi Ren, Xiaohang Yin, Siqi Wang, Yuwei Yan, Zhengyu Liu, Ming Wu, Dongchao Lu, Yan Yu, Lei Chen, Emeli Chatterjee, Guoping Li, Dragos Cretoiu, T Scott Bowen, Jin Li, Junjie Xiao**

Figure S1 to S15

Tables S1 to S3

Supporting Methods

Cells and treatment

C2C12 cells and 293T cells were prepared in DMEM (Corning, #10013100) containing 10% fetal bovine serum (FBS) (ExCell Bio, #FSP500) and 1% Penicillin-Streptomycin (KeyGen, #KGY0023) in standard incubator, by maintained environment at 37°C and 5% CO_2.

For inducing myotube formation, C2C12 myoblasts were plated into culture plates at a density of 40,000/ml. Then the differentiation medium, DMEM containing 2% horse serum (HS) (Gibco, #26050-088) and 1% Penicillin-Streptomycin, was used to promote differentiation for 4-5 days.

To establish the cellular muscle atrophy model, the mature myotubes were treated with 50 μM Dex (Sigma, #D4092) for 24h, 100 ng/ml TNFα (Sino Biological, #10602) or 500 nM AngII (Selleck, #P1085) for 48h.

Cell transfection

Transfection of C2C12 myotubes was performed by using Lipofectamine 2000 Reagent (Invitrogen, #11668019) according to standard method, that recommended by the manufacturer's instructions. The siRNAs of circDdb1 were synthesized by RiboBio (Guangzhou, China) and the transfection dose of siRNA was 50 nM. The sequences of used in this work are listed in Table S1. For knockdown of circDdb1 in muscle atrophy models, the transfection was performed first, and then muscle atrophy was induced after 24h.

qRT-PCR

Following the recommended procedure, Trizol (TaKaRa, #9019) was used to lyse and extract the total RNA from cells and tissues. The NanoDropTM One/OneC Microvolume UV-Vis Spectrophotometer (Thermo Scientific, #ND-ONE-W) was used to measure the RNA concentration. The Thermo Scientific™ RevertAid First Strand cDNA Synthesis Kit (Thermo Fisher, #K1622) was used to create cDNAs from the total RNA. The SYBR Green PCR kit (Takara, #RR820A) was then used for quantitative assays, with 18S rRNA used as an internal reference. The $2^{-\Delta\Delta Cq}$ technique was used to quantify the relative RNA levels. The primers used in qRT-PCR in this work are shown in Table S2 (Supporting Information).

To determinate the mitochondrial DNA copy number, the total DNA of C2C12 cells and muscle tissues was extracted and purified using the Tien Blood, cell and tissue Genomic DNA Extraction Kit (TIANGEN, #DP304). Then the total DNA was subjected to quantitative experiments by RT-PCR. The copy number of mt-Col represented the mitochondrial DNA copy number, and the copy number of GAPDH represented the genomic DNA copy number.

RNase R treatment

Total RNA samples were digested with RNase R solution (3U, Epicentre, #RNR07250) for 0 min, 5 min, 10 min, 15 min at 37°C, and 85°C for 5 min to inactivate the enzyme. The product was subjected to quantitative experiments by RT-PCR.

Nuclear and cytoplasmic RNA separation

PBS was used to collect and wash the C2C12 cells. After the cell precipitation was resuspended, it was lysed on ice for five minutes in 200 μL of lysis buffer A (10 mM Tris, pH=8.0, 1.5 mm MgCl2, 140 mM NaCl, 0.5% Nonidet P-40). The cytoplasmic supernatant was removed after 3 min of centrifugation at 1000×g, and 1 mL of Trizol lysis solution was added to extract the cytoplasmic RNAs. After that, lysis buffer A was used twice to wash the residual nuclear precipitate. The nuclear precipitate was then once again cleaned using lysis buffer A, which included 0.5% deoxycholic acid and 1% Tween-40. In order to extract nuclear RNA, the refined nuclear precipitate was then again suspended in 1 mL of Trizol solution. Nuclear and cytoplasmic RNA was extracted by Trizol (TaKaRa, #9019) according to standard protocol. Then the distribution of U1, GAPDH and circDdb1 were analyzed by qRT-PCR.

Fluorescence in situ hybridization (FISH)

The designed Cy3-labeled anti-sense-circDdb1 RNA probe and sense-random probe were synthesized by Sangon Biotech. At a density of 5,000/ml, C2C12 cells were seeded into μ-Slide 8 well glass plates (ibidi, #80826). After 4% paraformaldehyde (PFA) was used to fix the cells, 0.5% Triton X-100 (Sigma, #93443) with 2 mM ribonucleoside vanadyl complex (VRC) (NEB, #S1402S) was used to permeabilize the cells. They were then washed twice with 2×saline sodium citrate (SSC) buffer. The cells were then hybridized overnight at 37°C in a hybridization oven using hybridization buffer containing a probe (50 ng/ml). The next day, cells were washed with varying concentrations of SSC, added Cy3-labeled streptavidin, and allowed to keep at room temperature for one hour. After washing with PBS, nuclei were stained with DAPI (KeyGEN, #KGA215-50). Using a Zeiss laser scanning confocal microscope, images were captured at a magnification of 40×.

The following probes were used:

Sense-probe-for-circDdb1:

Biotin-5'-aaaAGTGCCAGCACCCAGATCACCTCAGCATCT-3';

Antisense-probe-for-circDdb1:

Biotin-5'-aaaAGATGCTGAGGTGATCTGGGTGCTGGCACT-3'.

Western blot

Cells and tissues were lysed in IP lysis buffer supplemented with phosphatase inhibitors and PMSF (KeyGen, #KGP9100). Equivalent proteins were separated by 10% SDS-PAGE gel and

then transferred to PVDF membranes (Pall Corporation, #BSP0161). Then the blocked membranes were probed with primary antibodies at 4°C overnight and incubation with appropriate HRP-conjugated secondary antibodies continuously. Protein bands were detected using Tanon's High-sig ECL Western Blotting Substrate (Tanon, #180-501) and BIO-RAD chemiluminescence equipment. Primary antibodies: p-mTOR (Ser-2448) (CST, #2971s), mTOR (CST, #2972s), p-FOXO3A (Ser-253) (CST,#9466s), FOXO3A (Abclonal, #A0102), p-p70S6K (CST, #9205), p70S6K (CST, #9202s), p-AKT (Ser-473) (CST, #4060), AKT (Proteintech, #10176-2-AP), p-EIF-4EBP1 (Abclonal, #AP0030), EIF4EBP1 (Abclonal, #A19045), BAX (Abclonal, #A19684), BCL2 (Abclonal, #A19693), Caspase3 (Abclonal, #A2156), Cleaved-caspase3 (Abclonal, #A11021), LC3A/LC3B (Abclonal, #A5618), P62 (Proteintech, #18420-1-AP), FLAG (Millipore, #F7425), DDB1 (Thermo #PA5-86064), and GAPDH (Bioworlde, #BS65529). Antibodies were stored and diluted according to manufacturer's instructions. For phosphorylated protein detection, membranes were stripped using rapid stripping solution before re-probing.

Immunofluorescence staining

Treated myotubes were rinsed with PBS, fixed in 4% PFA for 15 min, permeabilized with 0.5% Triton X-100 for 20 min, and blocked with 5% BSA. Cells were incubated with MF-20S primary antibody (DSHB, 1:100) for 2h at 4°C, then with Alexa Fluor 488 secondary antibody (Jackson ImmunoResearch, 1:200) for 2h at room temperature in the dark. Nuclei were visualized with Hoechst (KeyGEN, #KGA212-1). Images were collected by using a fluorescent microscope (Leica) and myotube diameter was quantified with ImageJ. For circDdb1-867aa staining, the FLAG primary antibody (Millipore, #F7425) was used.

WGA staining

OCT-embedded frozen muscles were cut into 10 μm sections, washed with PBS, fixed in 4% PFA for 30 min, and stained with WGA (1:100; Sigma, #L4895) for 2h at room temperature to visualize myofiber cross-sectional area (CSA). Nuclei were stained with Hoechst (1:2000; KeyGEN, #33342) for 20 min. Images were collected by using a fluorescence microscope (Zeiss). CSA was measured using ImageJ, with ≥500 fibers analyzed per mouse.

Muscle function tests

Grip strength was measured and recorded by grip strength meter (Yiyan Technology, #YLS-13A). Mice grasped the open pull bar and peak tension force was recorded. Each mouse was tested three times with 30s rest between trials and the average value was calculated. A blinded investigator conducted all analyses.

For treadmill running, mice were familiarized with the treadmill then run at a starting speed of 15 m/min, with 1 m/min increases every 4 min until exhaustion. Total running distance, speed and time were recorded.

Tetanic contraction force was assessed *ex vivo* using EDL muscles incubated in oxygenated Krebs buffer. Contractions were elicited and recorded using Dynamic Muscle Control software (Aurora Scientific, #ASI600A) with a 0.5s delay, 120 Hz frequency, and 0.3s duration.

Sucrose gradient fractionation assay

After transfection with the circDdb1 overexpression plasmid, C2C12 cells were incubated with 100 μg/mL cycloheximide (CHX) for 15 min before harvesting the cells. After washing with cold PBS twice, the cells were then harvested and lysed with 500 μL of lysis buffer (5 mM Tris-HCl, pH 7.5, 2.5 mM MgCl2, 1.5 mM KCl, 1 × protease inhibitor cocktail, 0.5% Triton X-100, 2 mM dithiothreitol (DTT), 0.5% sodium deoxycholate, 100 U of RNase inhibitor, and 100 mg/mL CHX) for 30 min. Afterwards, following centrifugation at 16,000×g for 10 min, the supernatant was recovered. To create a linearized gradient, the 5%-50% sucrose gradient solution was made in an ultracentrifuge tube and kept at 4°C overnight. After adding the supernatant to the sucrose gradient solution, the mixture was centrifuged for 3 hours at 100,000×g. Following centrifugation, the solution was successively poured from top to bottom into an EP tube in an amount of 150 μL, and the absorbance at 254 nm was measured using a UV spectrophotometer. Then, the RNA of each component was extracted and purified using the RNeasy Mini Kit (QIAGEN, #73404), followed by obtaining the cDNA by using a Thermo Scientific™ RevertAid First Strand cDNA Synthesis Kit (Thermo Fisher, #K1622). Then, circDdb1 was amplified with specific primers and analyzed by gel electrophoresis.

IRES site activity detection

The IRES sites of circDdb1 (IRES-1 125-270; IRES-2: 240-354) were cloned into TR-circGFP. Then the control plasmid, TR-circGFP, circDdb1-GFP-IRES-1 and circDdb1-GFP-IRES-2 were transfected into C2C12 cells inoculated in μ-Slide 8 well glass plates (ibidi, #80826). After 48h, cells were fixed with 4% PFA and stained with DAPI (KeyGEN, #KGA215-50). Subsequently, using a Carl Zeiss Confocal microscope (Zeiss, #LSM 800) with a 40× oil lens, the fluorescence signal and intensity of GFP were captured using identical photography settings.

As for the dual luciferase reporter assay, the protocol previously(19) outlined was followed. Both the full-length and truncated IRES sequences of circDdb1 were engineered into the P-Luc2-IRES-Report vector (Geneseed, Guangzhou, China). These vectors were transfected into C2C12 cells by the help of Lipofectamine 2000 Regent (Invitrogen, #11668019). After a 48-

hour incubation, the activities of firefly and Renilla luciferases were quantified using a dual-luciferase reporter assay kit (Promega, #E1910), adhering to the prescribed guidelines.

Plasmid construction

The plasmid skeleton used to construct cicDdb1 overexpression is pK5ssAAV-ciR (Geneseed Biotech, Guangzhou, China). The sequence of circDdb1 was synthesized by BGI (Shenzhen, China). Then the shRNA of circDdb1 was cloned into pENN.AAV.U6.ShRLuc.CMV.EGFP.SV40 plasmid, and the sequences used were listed as below.

circDdb1-shRNA-Forward:
(BamHI)5'-
GATCGCCAGCACCCAGATCACCTCTCGAGAGGTGATCTGGGTGCTGGCTTTTTG-3';
circDdb1-shRNA-Reverse:(EcoRI)5'-
AATTCAAAAAGCCAGCACCCAGATCACCTCTCGAGAGGTGATCTGGGTGCTGGC-3'.

The Flag-labeled circDdb1, with the 3×Flag sequence (GACTACAAAGACCATGACGGTGATTATAAAGATCATGACATCGATTACAAGGATGACGATGACAAG) inserted after the 189th nucleotide of circDdb1, was synthesized by GENEWIZ (Shenzhen, China), then cloned into pK5ssAAV-ciR (Geneseed Biotech, Guangzhou, China). The Flag-circDdb1-mut was modified based on Flag-circDdb1. In detail, a "T" was inserted at the 10th nucleotide of the open reading frame of circDdb1 to form a stop codon by means of site-directed mutagenesis. The kit used in the mutation experiments was the MutanBEST Kit (Takara, #R401), and the mutation primer sequences were listed below:
circDdb1-mut-Forward: 5'-CATTGAAGTGGCATGCTTGGATAT-3';
circDdb1-mut-Reverse: 5'-TTCCATCTTGTCATCGTCATCCTT-3'.

The IRES plasmid, with the two IRES sequences of circDdb1 IRES-1 (240-354) and IRES-2 (125-270), was synthesized by GENEWIZ (Shenzhen, China), and then cloned into TR-GFP. The Flag-circDdb1-867aa plasmid, with the sequence of Flag-circDdb1-867aa synthesized by BGI Tech (Shenzhen, China), was cloned into the Fugw plasmid (Addgene: #14883) between the BamHI and EcoRI sites.

AAV8 production and administration

HEK293T cells were seeded at $4×10^6$ cells/mL in 10 cm dishes and cultured at 37°C with 5% CO_2. At ~90% confluency, cells were transfected with a mixture of 10 μg pAAV2/8 (#112864), 10 μg pAdDeltaF6 (#112867), 10 μg pK5ssAAV-ciR-circDdb1 or control (pENN-sh-circDdb1

or control), and 90 μg PEI (1 mg/mL). Medium was refreshed 12h after transfection. Virus-containing cells and media were harvested 48h later. Media was centrifuged (4000 rpm, 30 min, 4°C) to pellet debris, mixed with 1/4 volume of PEG-8000, stirred for 1h, and incubated at 4°C overnight. Viral pellets were collected by centrifugation (4000 rpm, 30 min, 4°C), resuspended in lysis buffer (150 mM NaCl, 20 mM Tris pH 8.0), and subjected to three freeze-thaw cycles (-80°C/37°C) before centrifugation to collect the supernatant (4000 rpm, 30 min, 4°C). Supernatants were pooled, adjusted to 1 M $MgCl_2$, and digested with Benzonase (Merck, #101697, 250 U/mL) for 1h at 37°C. The virus was purified by iodixanol (Sigma, #1343517) gradient centrifugation, then titered and stored at -80°C until use.

For *in vivo* experiments, mice received intramuscular injections of AAV8-circDdb1 or AAV8-sh-circDdb1 into the gastrocnemius muscle at 1×10^{11} vg/mouse. Muscles were analyzed 6 weeks later (AAV8-circDdb1) or after 3 weeks (AAV8-sh-circDdb1) followed by 1 week of induced atrophy.

TUNEL staining

Muscle tissue slices that had been frozen were removed from the refrigerator at -80°C and thawed at room temperature for 30 min. In accordance with manufacturer's instructions, the DeadEnd fluorometric TUNEL system (Promega, #G3250) was used to measure the TUNEL staining. Briefly, the sections underwent 20 min of proteinase K (10 μg/mL) permeabilization, followed by a 10-min incubation period in an equilibration buffer. Following that, the sections were incubated for 1 hour in dark at 37°C by using rTdT incubation buffer. Lastly, Hoechst (1:2000; KeyGEN, #33342) was applied to the nuclei and let to keep at room temperature for 20 min. Using a Zeiss fluorescence microscope, the pictures were taken. The proportion of TUNEL-positive cell were measured using ImageJ software and at least 500 myofibers per mouse were examined.

Immunofluorescence identification of Muscle Fiber Types

Fresh muscle tissue samples embedded in OCT compound (−80°C) were sectioned into 15 μm-thick slices. Cryosections were fixed with 4% PFA for 10 min, followed by 0.25% Triton X-100 for 15 min, and 2 h at room temperature were spent blocking in 5% BSA. The sections were cleaned in PBS, then incubated overnight at 4°C with MHCI (1:3, DSHB, #BA-F8), MHCIIa (1:10, DSHB, #SC-71), and MHCIIb (1:3, DSHB, #BF-F3) and incubated with Alexa Fluor 350 anti-mouse IgG2b (1:200, Invitrogen, #A-21140), Alexa Fluor 488 anti-mouse IgG1 (1:200, Invitrogen, #A-21121) and Alexa Fluor 555 anti-mouse IgM (1:200, Invitrogen, #A-21426). Images were taken with a Zeiss fluorescence microscope using ImageJ software to calculate the ratio of muscle fibers that were MHCI-, MHCIIa-, and MHCIIb-positive.

Pulldown assays

After transfected with circDdb1 for 48 hours, the C2C12 cells were harvested. The cells were then resuspended in lysis buffer and placed on ice for 10 min. Following centrifugation (12000 g, 4°C, 10 min) of the cell lysates, the supernatant was separated into two fresh tubes. Double volumes of hybridization buffer were then added, along with 10 µg of Biotin-labeled circDdb1 probe or scramble probe, and 2 h at room temperature were spent incubating the mixture. After that, 100 µL Dynabeads TMMyOneTM Streptavidin T1 were added (Invitrogen, #65604D) and mixed on rotor for 1 h at room temperature. After washing the beads for 5 times with wash buffer the SDS-PAGE and protein silver staining using Rapid Silver dye kit (Beyotime, #P0017S) was performed to analyze the binding protein of circDdb1.

The probes used as follows:

Sense-probe-for-circDdb1: Biotin-5'-aaaAGTGCCAGCACCCAGATCACCTCAGCATCT-3';

Antisense-probe-for-circDdb1: Biotin-5'-aaaAGATGCTGAGGTGATCTGGGTGCTGGCACT-3'.

AGO2 RIP

Following a 48-hour transfection with circDdb1, C2C12 cells were lysed for 20 min on ice using IP lysis solution, which included protease inhibitors. The supernatant was collected following centrifugation at 12,000 rpm for 30 min at 4°C. The cell lysate supernatant was then separated into two tubes and treated separately for an overnight period at 4°C with rotation with an AGO2-specific antibody (Abcam, #ab186733) and a negative control IgG (Merck, #NI01). Then, DynabeadsTM Protein G was combined with the cell lysates for 2 h at 4°C. At last, extracted the RNA captured by beads by using the RNeasy Mini Kit (QIAGEN, #73404). To identify the enrichment of circDdb1, qRT-PCR was used.

Immunoprecipitation-mass spectrometry (IP/MS)

The protein coded by circDdb1 was identified by IP/MS. 48h after transfection with Flag-circDdb1, the C2C12 cells were lysed with IP lysis buffer for 20 min on ice. The supernatant was collected following 30 min of centrifugation at 12,000 rpm at 4°C. Subsequently, the cell lysate supernatant was separated into two tubes and incubated with with an anti-FLAG antibody (Sigma, #F3165) and IgG (Merck, #NI01). The next day, Dynabeads™ Protein G was combined with the cell lysates for 2 hours while rotating. After washing with IP lysis buffer for 5 times, the protein was dissolved with ultrapure water. Next, using a Rapid Silver dye kit (Beyotime, #P0017S), the target protein was identified by protein silver staining, and the target bands were cut for a mass spectrometry test carried out by GENECHEM Co., Ltd. (Shanghai, China).

Electrophoretic mobility shift (EMSA)

To conduct the RNA EMSA, we utilized the LightShift™ Chemiluminescent RNA EMSA Kit (Thermo Scientific, #20158). In this procedure, Biotin-labeled RNA probe was first heated to 80°C for 5 minutes and then promptly cooled on ice. The RNA probe (1nM) was then combined with 2μg of EIF4A3 fusion protein (Proteintech, #AG11130) and allowed to incubate in EMSA buffer for 30 minutes at room temperature. After incubation, the reactions were prepared with loading buffer and separated in a 5% BeyoGel™ TBE-Urea Precast PAGE Gel (Beyotime, #R0232S). The samples were then transferred onto a nylon membrane, which was crosslinked under UV light (254nm) for 5 minutes. Detection of the Biotin-labeled RNA was performed using the Chemiluminescent Nucleic Acid Detection Module (Thermo Scientific, #89880). The Biotin-labeled RNA probes were showed as following:

Probe	Sequence
regions A	Biotin-5’-aaa CCCAGATACTGAGTCATTGCTTTGTCAGTGTTATCTA ACTATTGGCCTGAAGACTGG-3’
regions B	Biotin-5’-aaa TATATAAACTCAATTGTGCTAGGTAAGATGAATGAATA GATAACAGTGTCAC-3’
regions C	Biotin-5’-aaa AATTTTGTAATCTTTACTAAGGAACTTTAAATAATTTAA ATGGCT-3’
regions D	Biotin-5’-aaa ATATTTTAGAGGTCTGATTTGGGGGTTGAGAAAAACTG CTAAGACTGGTGG-3’
regions E	Biotin-5’-aaa GCAGGACTTTGGGGAATAAGAAATAGAAGCCTCATGTA GCTTATTCGTAGGGCTCTATTAGGGCT-3’
regions F	Biotin-5’-aaa GGTCCTTTGTAGAGTGGTCGAATAGAGGAGGAACATGG GGTTTGGAGTCCGACAG-3’

<u>Supporting Figures</u>

```
Score            Expect   Identities      Gaps        Strand
1125 bits(609)   0.0      781/867(90%)    0/867(0%)   Plus/Plus

HSA_CIRC_0022284   ATCACTTCAGCATCGGTGAGGTTGGTCTCTCAAGAACCCAAAGCTCTGGTCAGTGAATGG   60
MMU_CIRC_0007604   ATCACCTCAGCATCTGTGAGGTTGGTGTCTCAAGAGCCCAAAGCTTTGGTCAGCGAGTGG   60
                   ***** ******** *********** ******** ********* ******* ** ***

HSA_CIRC_0022284   AAGGAGCCTCAGGCCAAGAACATCAGTGTGGCCTCCTGCAATAGCAGCCAGGTGGTGGTG   120
MMU_CIRC_0007604   AAAGAGCCTCAGGGCAAGAACATCAGTGTGGCCTCCTGTAACAGCAGCCAGGTGGTTGTT   120
                   ** ********** ************************ ** ************** **

HSA_CIRC_0022284   GCTGTAGGCAGGGCCCTCTACTATCTGCAGATCCATCCTCAGGAGCTCCGGCAGATCAGC   180
MMU_CIRC_0007604   GCCGTGGGAAGGGCACTATATTACCTTCAGATCCACCCTCAGGAGCTCCGGCAAATCAGC   180
                   ** ** ** ***** ** ** ** ** ******** ***************** ******

HSA_CIRC_0022284   CACACAGAGATGGAACATGAAGTGGCTTGCTTGGACATCACCCCATTAGGAGACAGCAAT   240
MMU_CIRC_0007604   CACACAGAGATGGAACATGAAGTGGCATGCTTGGATATCACCCCATTAGGGGACAGCAAT   240
                   ************************** ******** ************** *********

HSA_CIRC_0022284   GGACTGTCCCCTCTTTGTGCCATTGGCCTCTGGACGGACATCTCGGCTCGTATCTTGAAG   300
MMU_CIRC_0007604   GGCCTCTCCCCACTTTGTGCCATTGGACTTTGGACAGACATCTCAGCTCGTATCCTGAAG   300
                   ** ** ***** ************** ** ***** ******** ********* *****

HSA_CIRC_0022284   TTGCCCTCTTTTGAACTACTGCACAAGGAGATGCTGGGTGGAGAGATCATTCCTCGCTCC   360
MMU_CIRC_0007604   CTCCCATCCTTTGAACTACTACACAAGGAGATGCTGGGTGGAGAGATCATTCCTCGATCC   360
                    * ** ** *********** *********************************** ***

HSA_CIRC_0022284   ATCCTGATGACCACCTTTGAGAGTAGCCATTACCTCCTTTGTGCCTTGGGAGATGGAGCG   420
MMU_CIRC_0007604   ATCCTGATGACCACTTTTGAGAGTAGCCACTACCTCCTTTGTGCCTTGGGAGATGGGGCT   420
                   ************** ************** ************************** **

HSA_CIRC_0022284   CTTTTCTACTTTGGGCTCAACATTGAGACAGGTCTGTTGAGCGACCGTAAGAAGGTGACT   480
MMU_CIRC_0007604   CTTTTCTACTTTGGGCTCAACATCGAGACAGGTTTACTGAGTGACCGTAAAAAGGTGACT   480
                   *********************** ********* *  **** ******** *********

HSA_CIRC_0022284   TTGGGCACCCAGCCCACCGTATTGAGGACTTTTCGTTCTCTTTCTACCACCAACGTCTTT   540
MMU_CIRC_0007604   TTGGGCACCCAGCCCACGGTGTTGAGGACATTCCGTTCTCTTTCTACCACCAACGTCTTT   540
                   ***************** ** ******** ** ***************************

HSA_CIRC_0022284   GCTTGTTCTGACCGCCCCACTGTCATCTATAGCAGCAACCACAAATTGGTCTTCTCAAAT   600
MMU_CIRC_0007604   GCTTGCTCTGACCGGCCCACTGTCATCTATAGCAGCAATCACAAGTTGGTCTTCTCCAAT   600
                   ***** ******** *********************** ***** *********** ***

HSA_CIRC_0022284   GTCAACCTCAAGGAAGTGAACTACATGTGTCCCCTCAATTCAGATGGCTATCCTGACAGC   660
MMU_CIRC_0007604   GTCAACCTCAAGGAGGTGAACTATATGTGTCCTCTCAACTCAGATGGCTATCCTGACAGT   660
                   ************** ******** ******** ***** ********************

HSA_CIRC_0022284   CTGGCGCTGGCCAACAATAGCACCCTCACCATTGGCACCATCGATGAGATCCAGAAGCTG   720
MMU_CIRC_0007604   CTGGCATTGGCCAATAACAGCACTCTCACCATTGGCACCATCGATGAGATCCAGAAGCTC   720
                   *****  ******* ** ***** ***********************************

HSA_CIRC_0022284   CACATTCGCACAGTTCCCCTCTATGAGTCTCCAAGGAAGATCTGCTACCAGGAAGTGTCC   780
MMU_CIRC_0007604   CATATTCGCACAGTTCCCCTCTATGAGTCTCCCAGGAAGATCTGCTATCAGGAAGTGTCT   780
                   ** ***************************** ************** ***********

HSA_CIRC_0022284   CAGTGTTTCGGGGTCCTCTCCAGCCGCATTGAAGTCCAAGACACGAGTGGGGGCACGACA   840
MMU_CIRC_0007604   CAGTGCTTTGGGGTCCTTTCCAGCCGCATTGAAGTCCAAGATAGCAGTGGAGGCACTACT   840
                   ***** ** ******** *********************** *  ***** ***** **

HSA_CIRC_0022284   GCCTTGAGGCCCAGCGCTAGCACCCAG   867
MMU_CIRC_0007604   GCTTTGAGGCCCAGTGCCAGCACCCAG   867
                   ** *********** ** *********
```

Figure S1. circDdb1 (circBase ID: mmu_circ_0007604) was conserved cross human (circBase ID: hsa_circ_0022284) and mouse species.

Sequence alignment of *mmu*_circDdb1 and *hsa*_circDdb1.

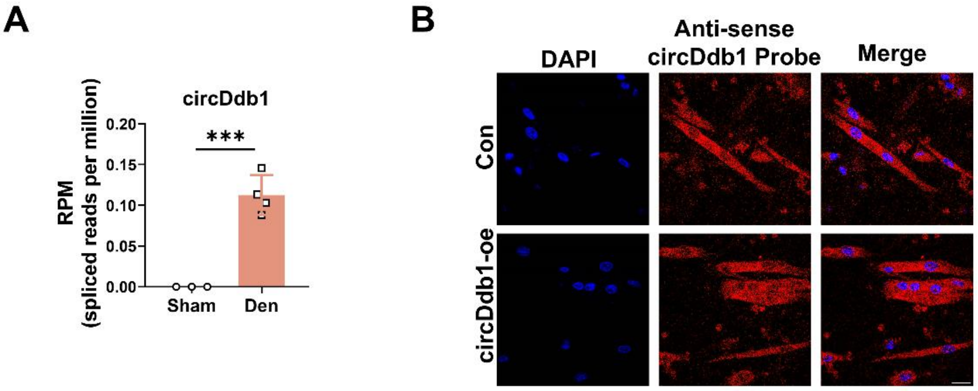

C

Donor	Gender	Age	Height (m)	BW (kg)	Group
Donor1	M	25	1.65	65	Young
Donor2	M	32	1.77	65	
Donor3	F	29	1.55	62	
Donor4	M	30	1.72	66	
Donor5	M	15	1.68	55	
Donor6	F	72	1.65	68	Aging
Donor7	F	67	1.56	65	
Donor8	M	72	1.67	79	
Donor9	M	80	1.62	62	
Donor10	M	77	1.7	83	

Figure S2. Expression of circDdb1 in muscle atrophy.

A. RPM (spliced reads per million) value of circDdb1 in RNA sequencing results (n=3-4 per group, GSE205537). B. Representative images of FISH showing the distribution of circDdb1 in C2C12 myotubes (scale bar: 40 μm). B. The information of muscles sample donors. Statistical analysis was performed using an unpaired, two-tailed Student's t-test to compare between two groups. ***$p < 0.001$. Data are represented as mean ± SD.

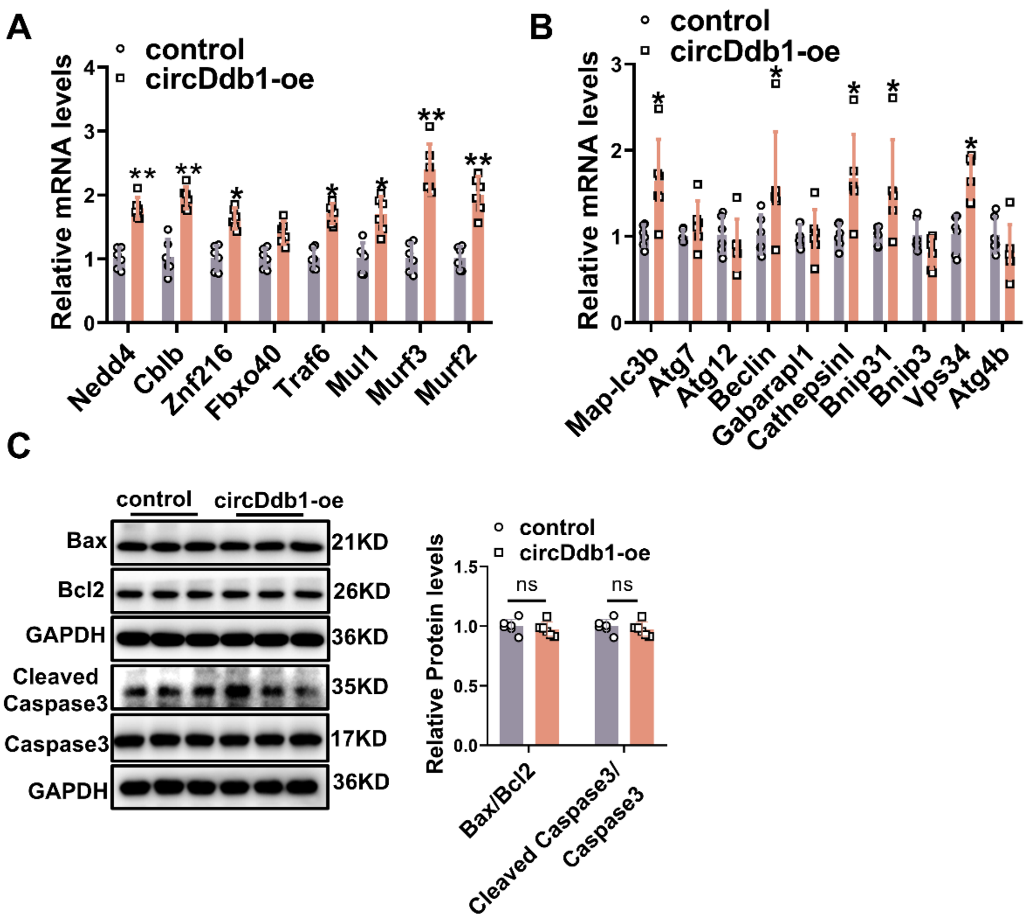

Figure S3. circDdb1 induces muscle atrophy *in vitro*.

A. qRT-PCR assessing UPS-related gene expression levels in C2C12 myotubes transfected with circDdb1-oe and control (n=6). B. qRT-PCR assessing autophagy-related gene expression levels in C2C12 myotubes transfected with circDdb1-oe and control (n=6). C. Western blot assessing Bax, Bcl2, and Caspase3 protein expression levels in C2C12 myotubes transfected with circDdb1-oe and control (n=6). Statistical analysis was performed using an unpaired, two-tailed Student's t-test to compare between two groups. Data are represented as mean ± SD. *p < 0.05; **p < 0.01. circDdb1-oe: circDdb1 overexpression plasmid; control: control plasmid.

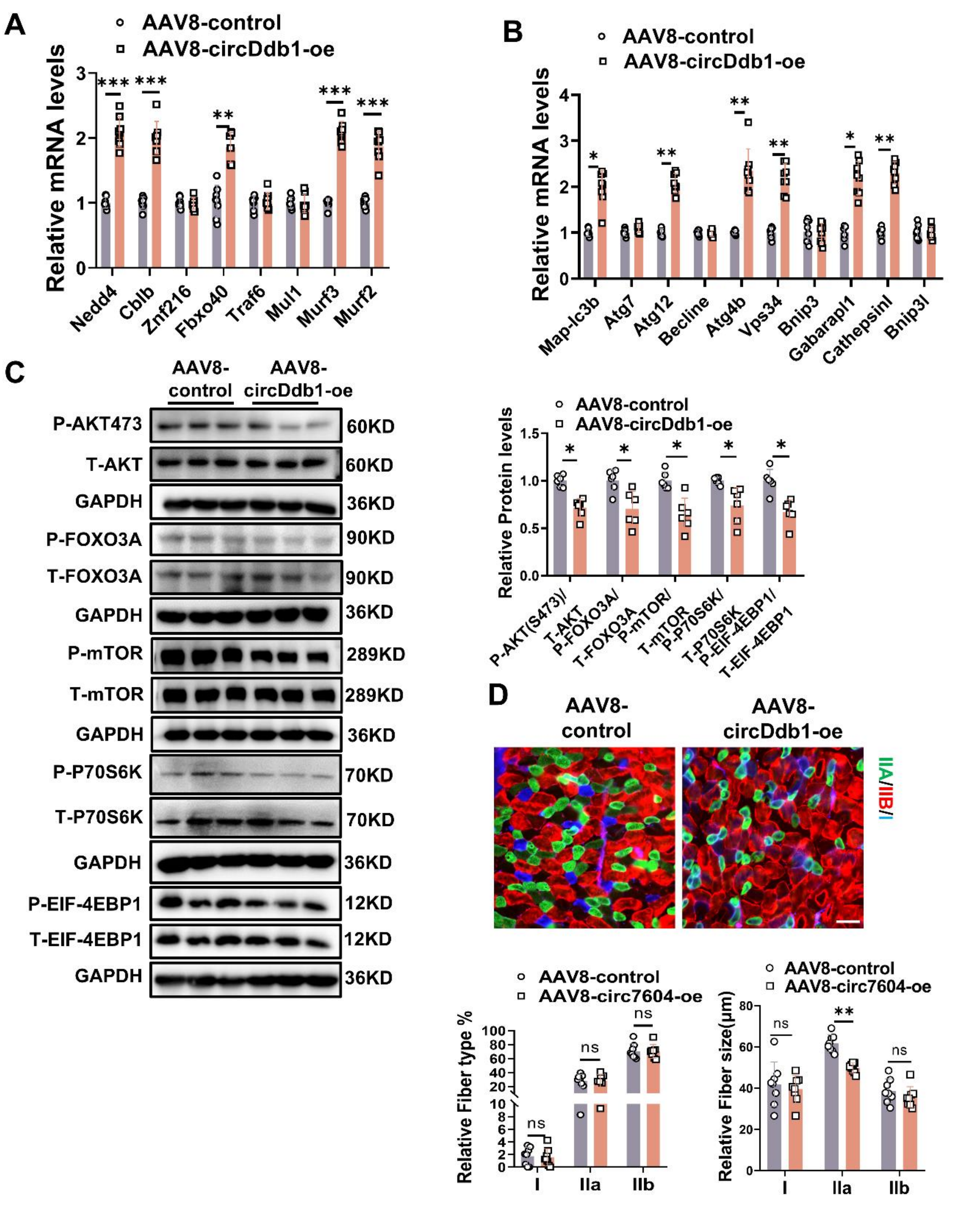

Figure S4. circDdb1 induces muscle atrophy *in vivo*.

A. qRT-PCR assessing UPS-related gene expression levels in gastrocnemius muscle of mice injected with AAV8-circDdb1-oe and AAV8-control (n=8). B. qRT-PCR assessing autophagy-related expression gene levels in gastrocnemius muscle of mice injected with AAV8-circDdb1-oe and AAV8-control (n=8). C. Western blot assessing AKT/FOXO3A/mTOR pathway related protein expression levels (including p-AKT, p-FOXO3A, p-mTOR, p-P70S6K, p-EIF-4EBP1) in gastrocnemius muscle of mice injected with AAV8-circDdb1-oe and AAV8-control (n=6). D. Immunofluorescence staining assessing fine fiber types in gastrocnemius muscle of mice

injected with AAV8-circDdb1-oe and AAV8-control (n=8), scale bar: 100 μm. Statistical analysis was performed using an unpaired, two-tailed Student's t-test to compare between two groups. Data are represented as mean ± SD. *p < 0.05; **p < 0.01; ***p < 0.001. AAV8-circDdb1-oe: circDdb1 overexpression adeno-associated virus 8; AAV8-control: control AAV8.

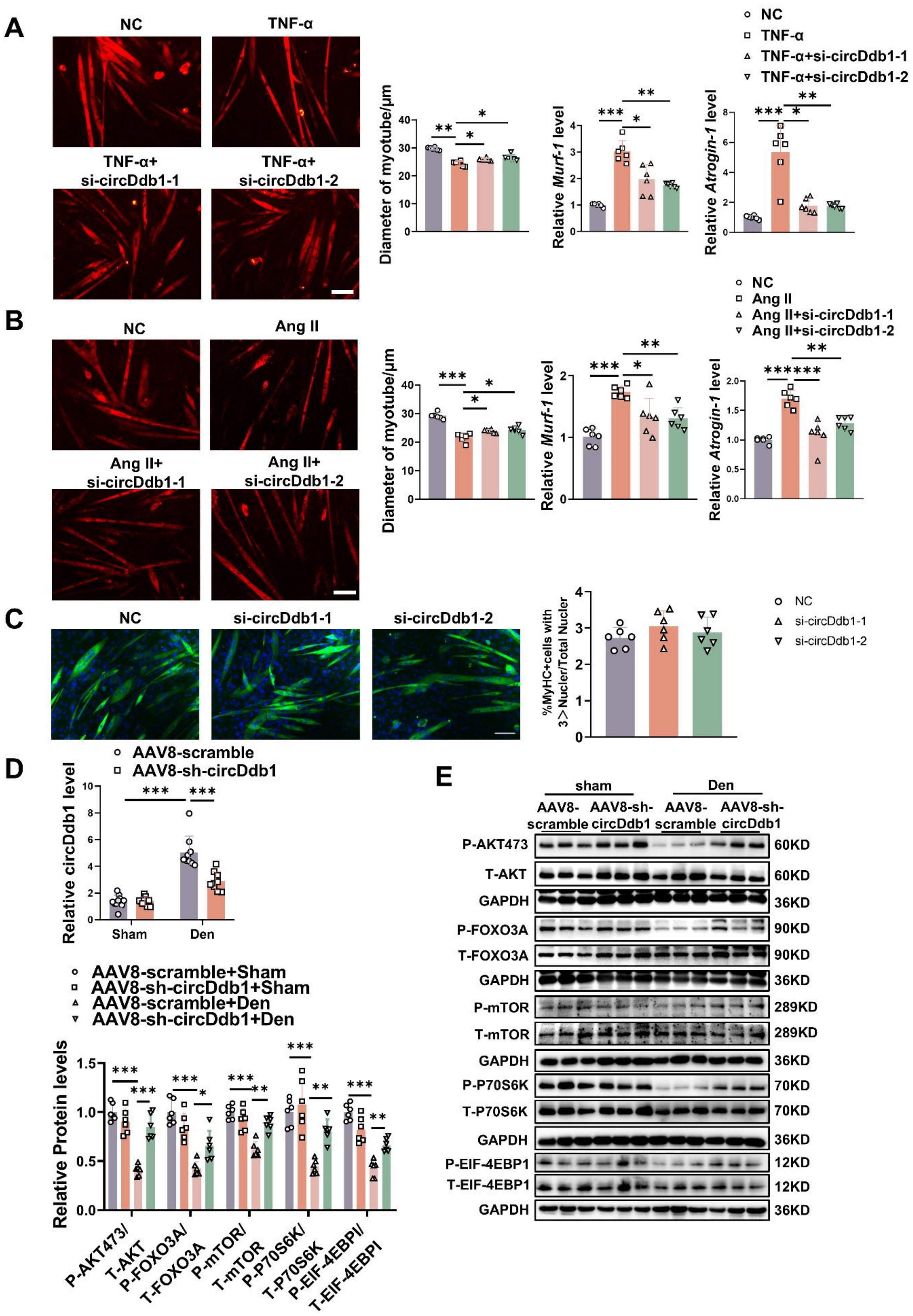

Figure S5. Downregulation of circDdb1 expression mitigates muscle atrophy both *in vitro* and *in vivo*.

A. Representative images and statistical analysis of the diameter of C2C12 myotubes, and qRT-PCR assessment of MuRF-1 and Atrogin-1 gene expression levels in C2C12 myotubes transfected with si-circDdb1 in model of TNF-α-treated muscle atrophy ($n = 6$), scale bar: 100 μm. B. Representative images and statistical analysis of the diameter of C2C12 myotubes, and qRT-PCR assessment of MuRF-1 and Atrogin-1 gene expression levels in C2C12 myotubes transfected with si-circDdb1 in model of Angiotensin II (Ang II)-treated muscle atrophy ($n = 6$), scale bar: 100 μm. C. Representative images and statistical analysis of the differentiation from C2C12 myoblasts to C2C12 myotubes ($n = 6$), scale bar: 50 μm. D. qRT-PCR assessing circDdb1 expression level in gastrocnemius muscle of mice injected with AAV8-sh-circDdb1 in model of denervation (Den)-induced muscle atrophy ($n = 10, 10, 9, 9$). E. Western blot assessing AKT/FOXO3A/mTOR pathway related protein expression levels (including p-AKT, p-FOXO3A, p-mTOR, p-P70S6K, p-EIF-4EBP1) in mice injected with AAV8-sh-circDdb1 in model of denervation (Den)-induced muscle atrophy ($n = 6$). Statistical analysis was conducted employing a one-way ANOVA test followed by Bonferroni test for A-C, and a two-way ANOVA test followed by Tukey post hoc test involving D and E. Data are represented as mean ± SD. $^{*}p < 0.05$; $^{**}p < 0.01$; $^{***}p < 0.001$. si-circDdb1: small interfering RNA against circDdb1(including si-circDdb1-1 and si-circDdb1-2); NC: negative control small interfering RNA. AAV8-sh-circDdb1: circDdb1 knockdown adeno-associated virus 8; AAV8-scramble: control AAV8.

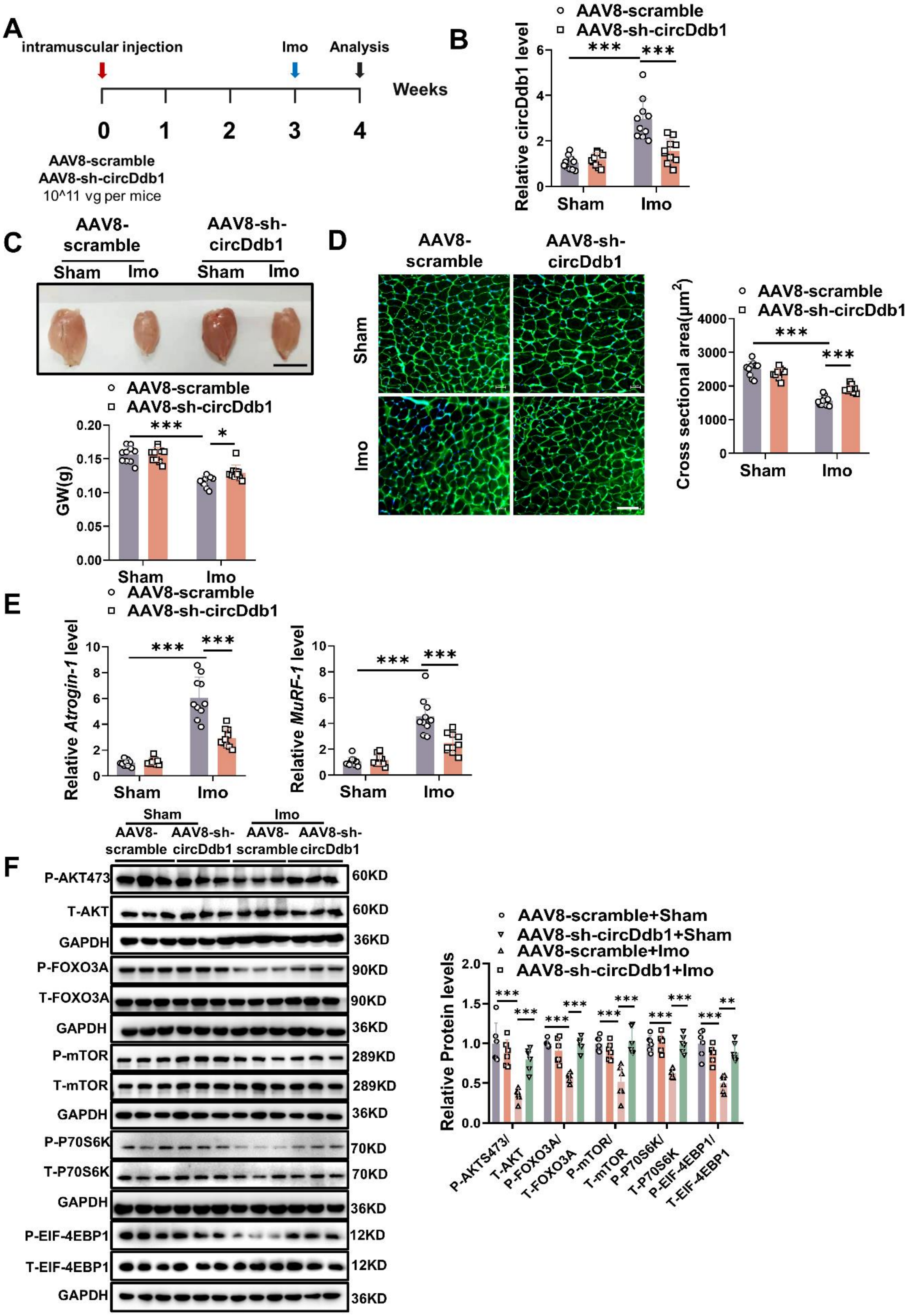

Figure S6. Suppression of circDdb1 mitigates muscle atrophy induced by immobilization *in vivo*.

A. Schematic representation of the experimental design for viral injection and the construction of the model of muscle atrophy induced by immobilization (Imo). B. qRT-PCR assessing circDdb1 expression level in gastrocnemius muscle of mice injected with AAV8-sh-circDdb1 in model of immobilization (Imo)-induced muscle atrophy (n = 10). C. Representative images of gastrocnemius muscle morphology and gastrocnemius muscle weight (GW) of mice injected with AAV8-sh-circDdb1 in model of immobilization (Imo)-induced muscle atrophy (n = 10), scale bar: 1 cm. D. Representative images and statistical analysis of myofiber cross-sectional area of mice injected with AAV8-sh-circDdb1 in model of immobilization (Imo)-induced muscle atrophy (n = 10), scale bar: 100 μm. E. qRT-PCR assessing MuRF-1 and Atrogin-1 gene expression levels in gastrocnemius muscle of mice injected with AAV8-sh-circDdb1 in model of immobilization (Imo)-induced muscle atrophy (n = 10). F. Western blot assessing AKT/FOXO3A/mTOR pathway related protein expression levels (including p-AKT, p-FOXO3A, p-mTOR, p-P70S6K, p-EIF-4EBP1) in mice injected with AAV8-sh-circDdb1 in model of immobilization (Imo)-induced muscle atrophy (n = 6). Statistical analysis was performed using two-way ANOVA with Tukey test to compare between multiple groups. Data are represented as mean ± SD. *p < 0.05; **p < 0.01; ***p < 0.001. AAV8-sh-circDdb1: circDdb1 knockdown adeno-associated virus 8; AAV8-scramble: control AAV8.

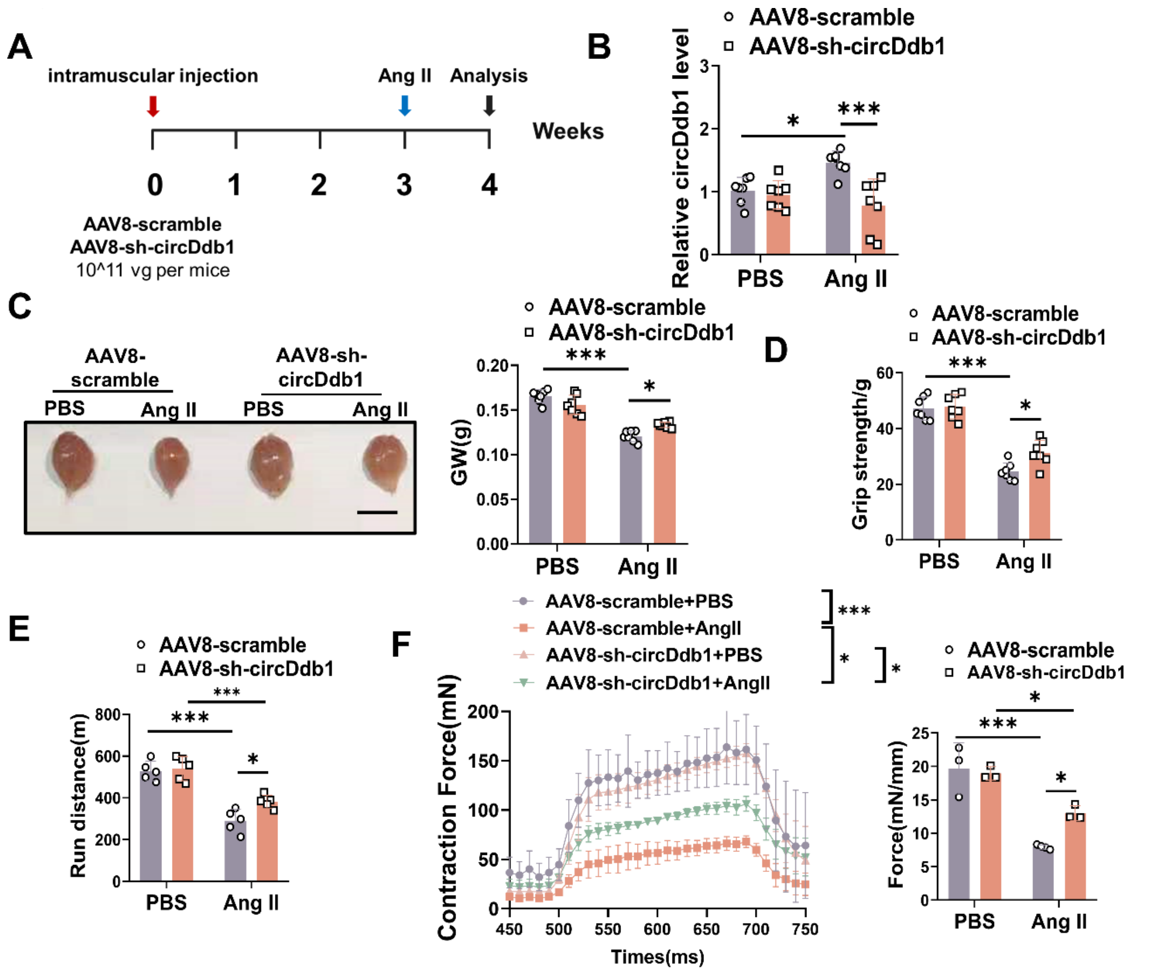

Figure S7. Suppression of circDdb1 mitigates muscle atrophy induced by Angiotensin II *in vivo*.

A. Schematic representation of the experimental design for viral injection and the construction of the model of muscle atrophy induced by Angiotensin II (AngII). B. qRT-PCR assessing circDdb1 expression level in gastrocnemius muscle of mice injected with AAV8-sh-circDdb1 in model of Angiotensin II (AngII)-induced muscle atrophy (n = 7). C. Representative images of gastrocnemius muscle morphology and gastrocnemius muscle weight (GW) of mice injected with AAV8-sh-circDdb1 in model of angiotensin II (Ang II)-induced muscle atrophy (n = 7), scale bar: 1 cm. D. Statistical analysis of grip strength in the right hind limb muscles of mice injected with AAV8-sh-circDdb1 in the model of angiotensin II (Ang II)-induced muscle atrophy (n = 7). E. Statistical analysis of running distance in the right hind limb muscles of mice injected with AAV8-sh-circDdb1 in the model of angiotensin II (Ang II)-induced muscle atrophy (n=4-5). F. Statistical analysis of EDL muscle contraction force of mice injected with AAV8-sh-circDdb1 in the model of angiotensin II (Ang II)-induced muscle atrophy (n=3-4). Statistical analysis was performed using two-way ANOVA with Tukey test to compare between

multiple groups. Data are represented as mean ± SD. *$p < 0.05$; ***$p < 0.001$. AAV8-sh-circDdb1: circDdb1 knockdown adeno-associated virus 8; AAV8-scramble: control AAV8.

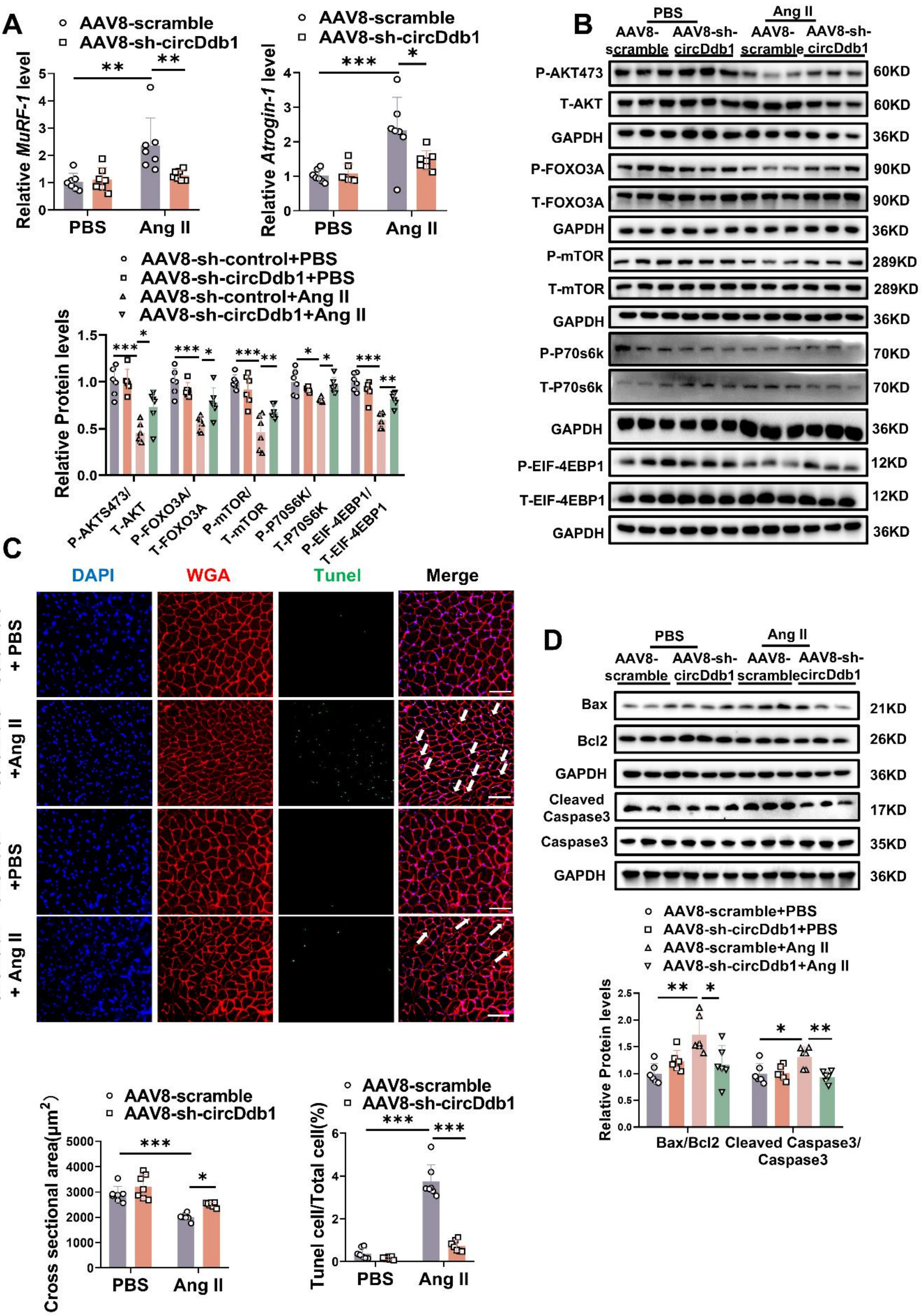

Figure S8. Suppression of circDdb1 mitigates muscle atrophy induced by Angiotensin II *in vivo*.

A. qRT-PCR assessing MuRF-1 and Atrogin-1 gene expression levels in gastrocnemius muscle of mice injected with AAV8-sh-circDdb1 in model of angiotensin II (Ang II)-induced muscle atrophy (n =7). B. Western blot assessing AKT/FOXO3A/mTOR pathway related protein expression levels (including p-AKT, p-FOXO3A, p-mTOR, p-P70S6K, p-EIF-4EBP1) in mice injected with AAV8-sh-circDdb1 in model of angiotensin II (Ang II)-induced muscle atrophy (n = 6). C. Representative images and statistical analysis of myofiber cross-sectional area and quantification of TUNEL-positive cells in mice injected with AAV8-sh-circDdb1 in model of angiotensin II (Ang II)-induced muscle atrophy (n = 7), scale bar: 100μm. D. Western blot assessing Bax, Bcl2 and Caspase3 protein expression levels in mice injected with AAV8-sh-circDdb1 in model of angiotensin II (Ang II)-induced muscle atrophy (n = 6). Statistical analysis was performed using two-way ANOVA with Tukey test to compare between multiple groups. Data are represented as mean ± SD. $^{**}p < 0.01$; $^{***}p < 0.001$. AAV8-sh-circDdb1: circDdb1 knockdown adeno-associated virus 8; AAV8-scramble: control AAV8.

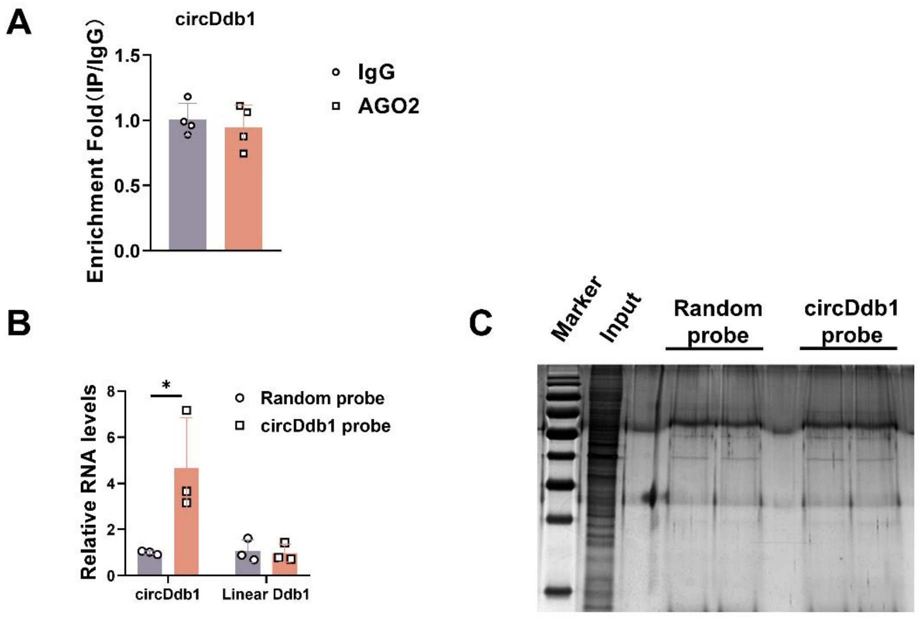

Figure S9. CircDdb1 does not act as miRNA sponge and binding protein.

A. qRT-PCR assessing circDdb1 expression level in the immunoprecipitation of AGO2 from the C2C12 cells (n=3). B. qRT-PCR assessing circDdb1 and Ddb1 expression levels in the pulldown of biotin-labeled circDdb1 probe from the C2C12 cells (n=3). C. Silver staining of biotin-labeled circDdb1 probe pulldown. Statistical analysis was performed using an unpaired, two-tailed Student’s t-test to compare between two groups. *$p < 0.05$. Data are represented as mean ± SD.

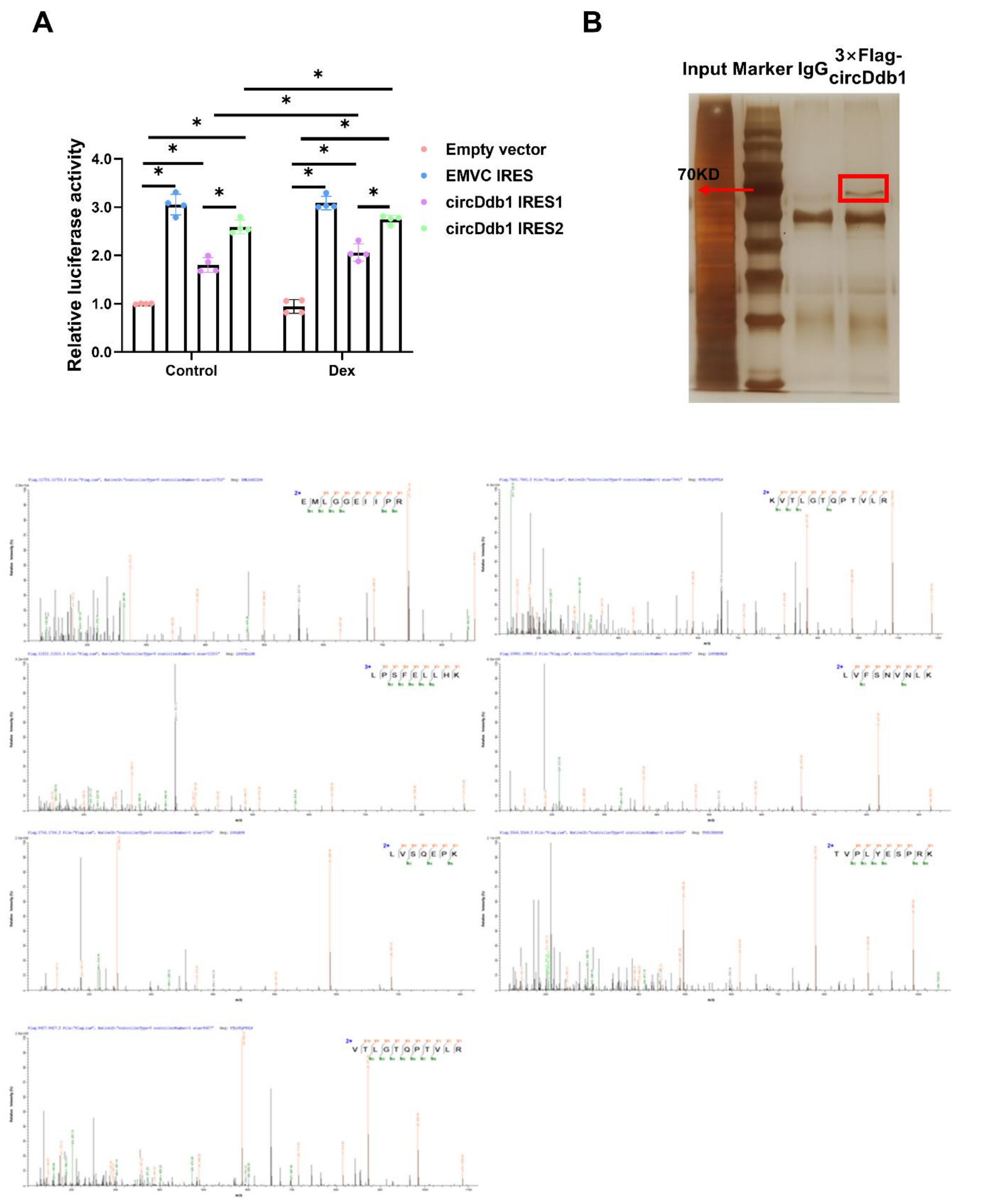

Figure S10. The translation ability of circDdb1.

A. Luc/Rluc activities in C2C12 myotube of each transfected plasmid in baseline and dexamethasone treatment (n=4). B. Silver staining detection of immunoprecipitated samples with anti-Flag antibodies, and seven specific peptide fragments of circDdb1-867aa detected by mass spectrometry. Statistical analysis was performed using two-way ANOVA with Tukey test

to compare between multiple groups (A). **$p < 0.01$; ***$p < 0.001$. Data are represented as mean ± SD.

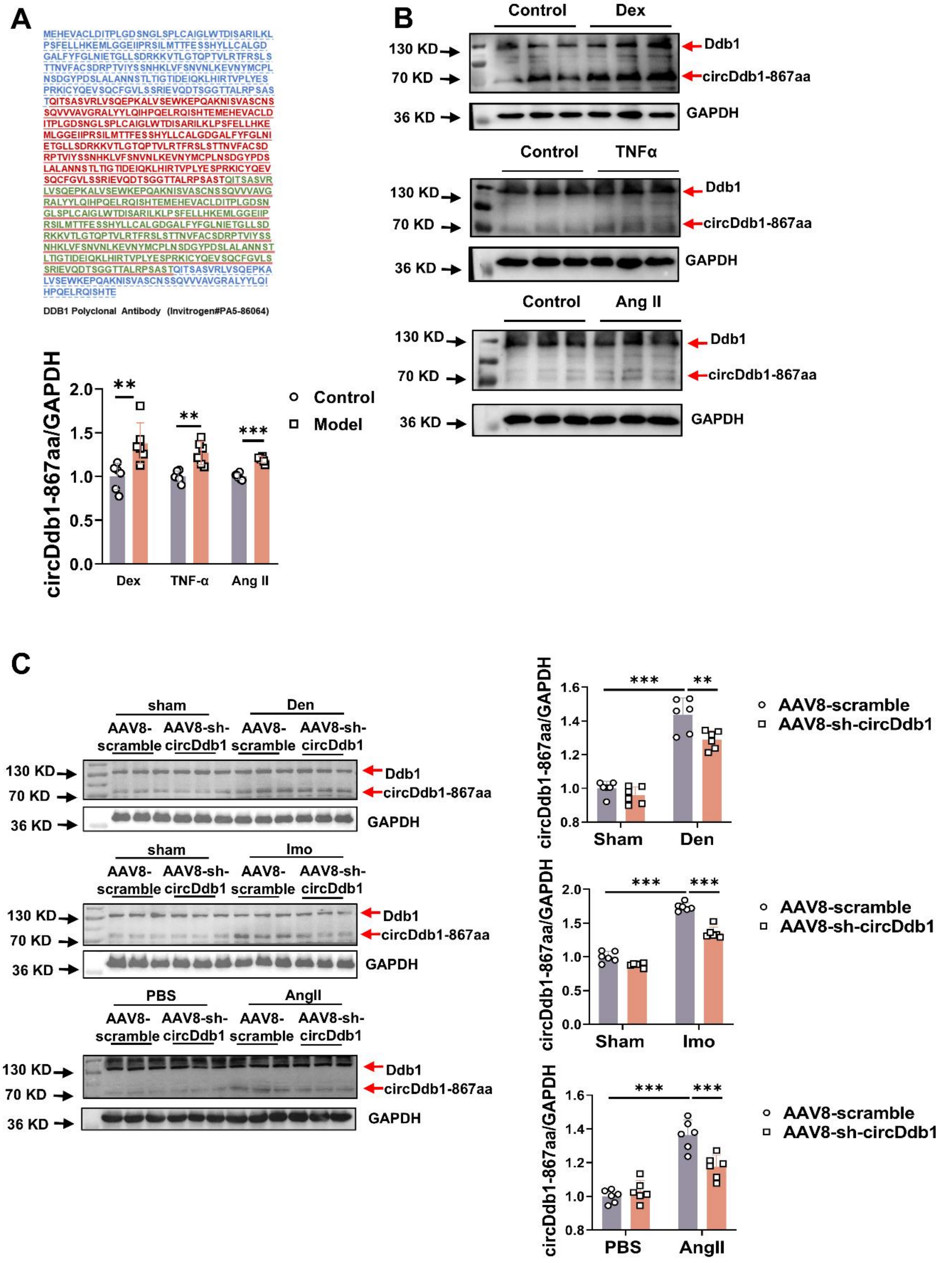

Figure S11. Detection of circDdb1-867aa.

A. An antibody against DDB1 recognized the predicted circDdb1-867aa peptide. The amino acid sequences labeled in red, blue, and green were the recognition sites for the anti-DDB1

antibody (Invitrogen #PA5-86064). B. Western blot analysis of circDdb1-867aa expression in cultured C2C12 myotubes exposed to Dex, TNF-α and AngII (n = 6). C. Western blot analysis of circDdb1-867aa expression in mice injected with AAV8-sh-circDdb1 in model of Den, Imo AngII-induced muscle atrophy (n = 6). Statistical analysis was conducted employing an unpaired, two-tailed Student's t-test for B, and a two-way ANOVA test followed by Tukey post hoc test for C. $**p < 0.01$; $***p < 0.001$. Data are represented as mean ± SD.

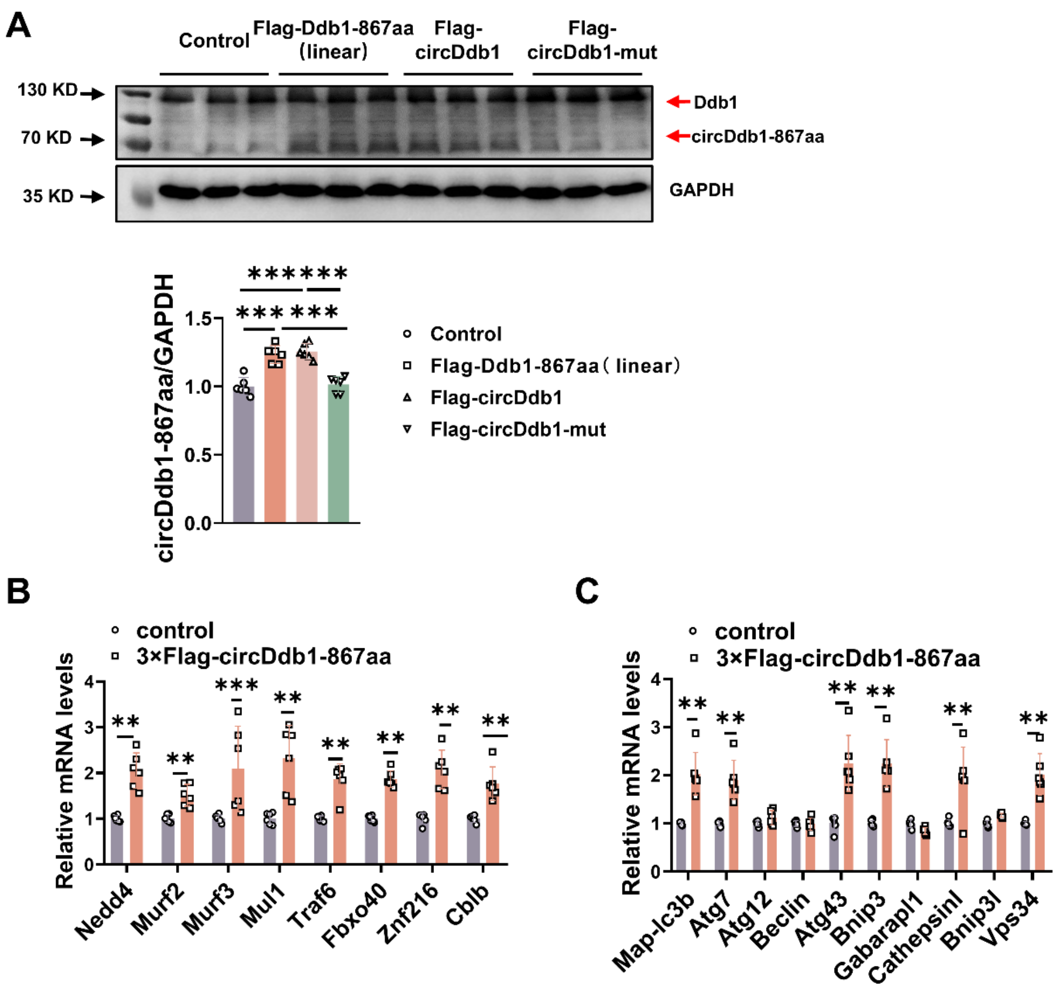

Figure S12. circDdb1-867aa protein induces muscle atrophy *in vitro*.

A. Western blot assessing circDdb1-867aa protein expression level in C2C12 myotubes transfected with Fugw-circDdb1-867aa, Flag-circDdb1 and Flag-circDdb1-mut plasmid (n = 6). B. qRT-PCR assessing UPS-related gene expression levels in C2C12 myotubes transfected with Fugw-3×Flag-circDdb1-867aa (n = 6). C. qRT-PCR assessing autophagy-related gene expression levels in C2C12 myotubes transfected with Fugw-3×Flag-circDdb1-867aa (n = 6). Statistical analysis was conducted employing a one-way ANOVA test followed by Bonferroni test for A, and an unpaired, two-tailed Student's t-test for B and C. **p < 0.01; ***p < 0.001. Data are represented as mean ± SD.

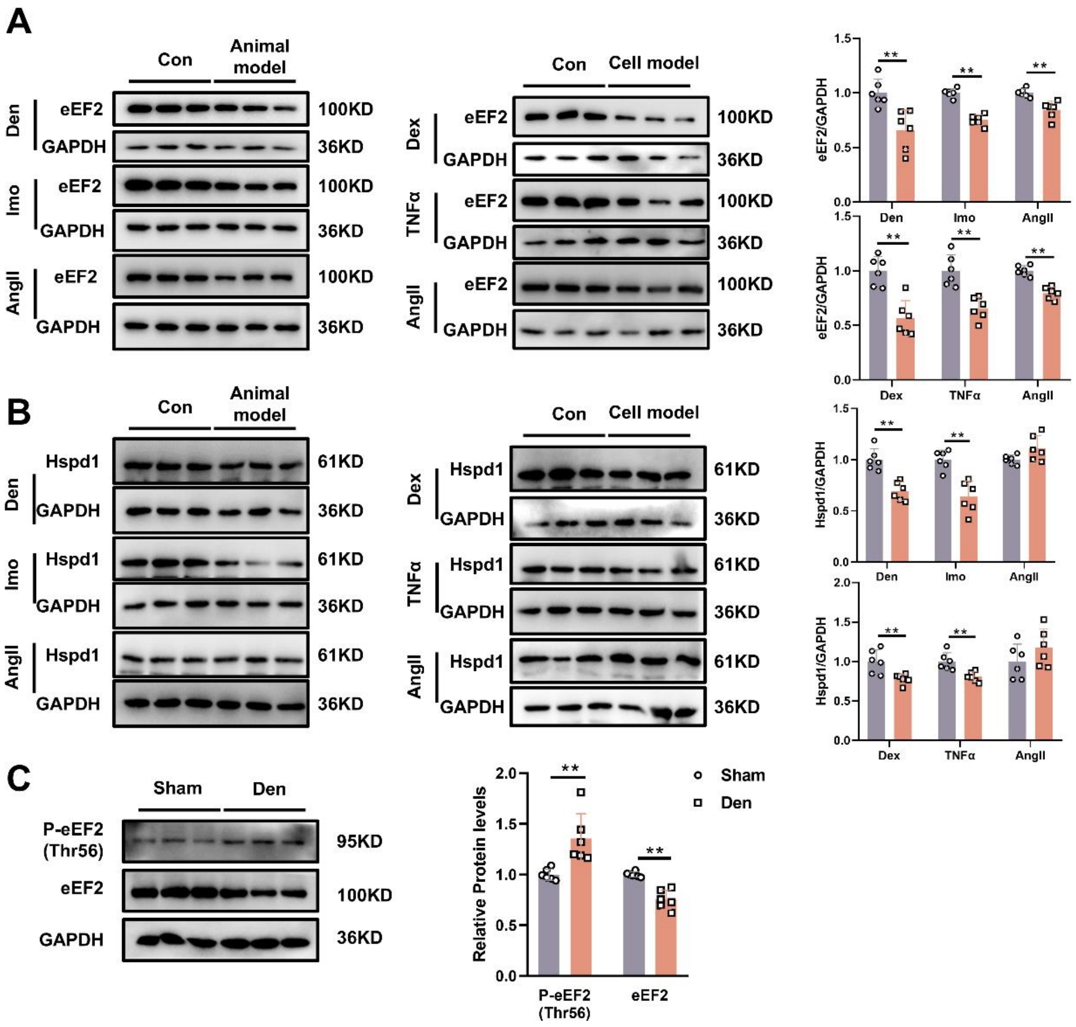

Figure S13. eEF2 is downregulated in the muscle atrophy model, while p-eEF2(Thr56) is upregulated in the muscle atrophy model.

A. Western blot assessing eEF2 proteins expression level in animal muscle atrophy models (including Den-, Imo-, and AngII-induced muscle atrophy models) and cell muscle atrophy models (including Dex, TNF-α and AngII-treated C2C12 myotubes) (n = 6). B. Western blot assessing Hspd1 protein expression level in animal muscle atrophy models (including Den-, Imo-, and AngII-induced muscle atrophy models) and cell muscle atrophy models (including Dex, TNF-α and AngII-treated C2C12 myotubes) (n = 6). C. Western blot assessing P-eEF2 (Thr56) and eEF2 protein expression levels in Den-induced muscle atrophy model (n = 6). Statistical analysis was performed using an unpaired, two-tailed Student's t-test to compare between two groups. $**p < 0.01$. Data are represented as mean ± SD.

Figure S14

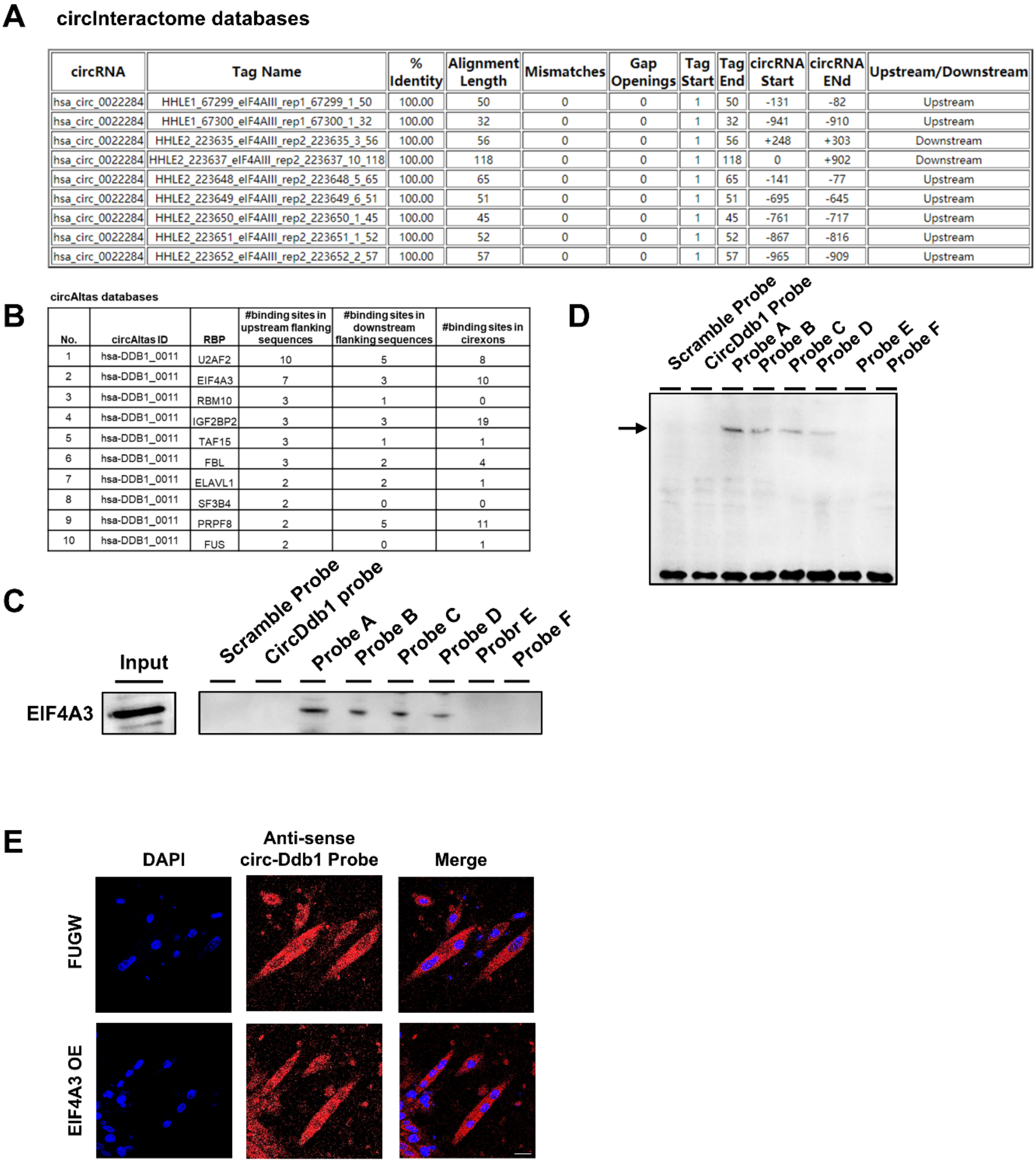

circRNA	Tag Name	% Identity	Alignment Length	Mismatches	Gap Openings	Tag Start	Tag End	circRNA Start	circRNA ENd	Upstream/Downstream
hsa_circ_0022284	HHLE1_67299_eIF4AIII_rep1_67299_1_50	100.00	50	0	0	1	50	-131	-82	Upstream
hsa_circ_0022284	HHLE1_67300_eIF4AIII_rep1_67300_1_32	100.00	32	0	0	1	32	-941	-910	Upstream
hsa_circ_0022284	HHLE2_223635_eIF4AIII_rep2_223635_3_56	100.00	56	0	0	1	56	+248	+303	Downstream
hsa_circ_0022284	HHLE2_223637_eIF4AIII_rep2_223637_10_118	100.00	118	0	0	1	118	0	+902	Downstream
hsa_circ_0022284	HHLE2_223648_eIF4AIII_rep2_223648_5_65	100.00	65	0	0	1	65	-141	-77	Upstream
hsa_circ_0022284	HHLE2_223649_eIF4AIII_rep2_223649_6_51	100.00	51	0	0	1	51	-695	-645	Upstream
hsa_circ_0022284	HHLE2_223650_eIF4AIII_rep2_223650_1_45	100.00	45	0	0	1	45	-761	-717	Upstream
hsa_circ_0022284	HHLE2_223651_eIF4AIII_rep2_223651_1_52	100.00	52	0	0	1	52	-867	-816	Upstream
hsa_circ_0022284	HHLE2_223652_eIF4AIII_rep2_223652_2_57	100.00	57	0	0	1	57	-965	-909	Upstream

No.	circAltas ID	RBP	#binding sites in upstream flanking sequences	#binding sites in downstream flanking sequences	#binding sites in cirexons
1	hsa-DDB1_0011	U2AF2	10	5	8
2	hsa-DDB1_0011	EIF4A3	7	3	10
3	hsa-DDB1_0011	RBM10	3	1	0
4	hsa-DDB1_0011	IGF2BP2	3	3	19
5	hsa-DDB1_0011	TAF15	3	1	1
6	hsa-DDB1_0011	FBL	3	2	4
7	hsa-DDB1_0011	ELAVL1	2	2	1
8	hsa-DDB1_0011	SF3B4	2	0	0
9	hsa-DDB1_0011	PRPF8	2	5	11
10	hsa-DDB1_0011	FUS	2	0	1

Figure S14. EIF4A3 binding the flanking regions of circDdb1.

A-B. The RBP of flanking sequences of circDdb1 were predicted using CircInteractome and circAltas databases. C. Precipitation obtained by RNA pulldown assay using the probes of putative sites, and western blot analysis for EIF4A3. D. Electrophoretic mobility shift (EMSA) assay of the direct association between EIF4A3 protein and the probes of putative sites. Arrow denotes the formation of the RNA/protein-binding complex. E. Representative images of FISH showing the distribution of circDdb1 in C2C12 myotube treated with EIF4A3 OE plasmid (scale bar: 40 μm).

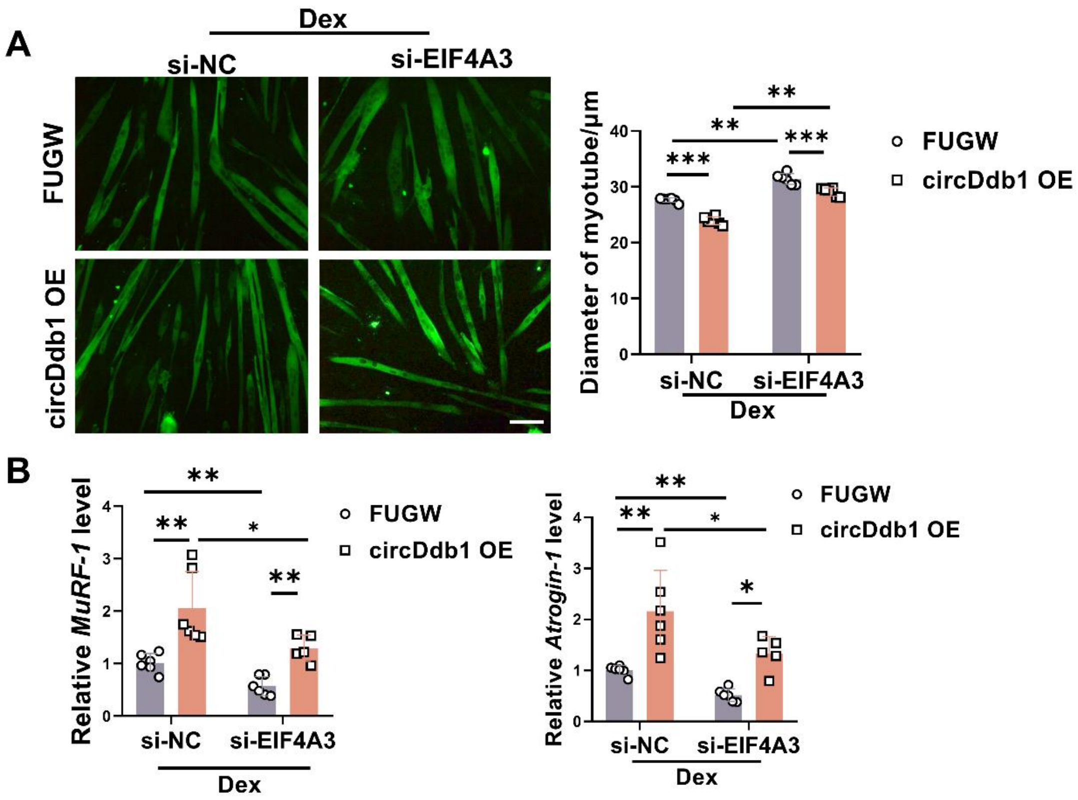

Figure S15. EIF4A3 induces circDdb1 expression in muscle atrophy.

A. Representative images and statistical analysis of myotubes diameter in C2C12 myotubes transfected with circDdb1 OE and si-EIF4A3 in dexamethasone (Dex)-induced muscle atrophy model (n = 6), scale bar: 100 μm. B. qRT-PCR assessing MuRF-1 and Atrogin-1 gene expression levels in C2C12 myotubes transfected with circDdb1 OE and si-EIF4A3 in dexamethasone (Dex)-induced muscle atrophy model (n = 6). Statistical analysis was performed using two-way ANOVA with Tukey test to compare between multiple groups. *p < 0.05; **p < 0.01; ***p < 0.001. Data are represented as mean ± SD. circDdb1 OE: circDdb1 overexpression lentiviruses; Fugw: control lentivirus. si-EIF4A3: small interfering RNA targeting circDdb1; si-Nc: small interfering RNA negative control.

Supporting Tables

Table S1. siRNA sequences used in this work

**siRNA**	**Sequence**
siRNA-circDdb1-1	CACCCAGAUCACCUCAGCA<br>UGCUGAGGUGAUCUGGGUG
siRNA-circDdb1-2	CAGCACCCAGAUCACCUCA<br>UGAGGUGAUCUGGGUGCUG
siRNA-circDdb1-3	GCCAGCACCCAGAUCACCU<br>AGGUGAUCUGGGUGCUGGC
siRNA-eif4a3-1	UAAUUAUCUGCUUGAUAGCAC<br>GCUAUCAAGCAGAUAAUUAAA
siRNA-eif4a3-1	UUUCCAUUCCUCUCUUUCCAC<br>GGAAAGAGAGGAAUGGAAAUU

Table S2. Primer sequences used in this work

Gene	Forward（5'-3'）	Reverse（5'-3'）
mmu-18s	TCAAGAACGAAAGTCGGAGG	GGACATCTAAGGGCATCAC
mmu-Murf-1	GTGTGAGGTGCCTACTTGCTC	GCTCAGTCTTCTGTCCTTGGA
muu-Atrogin-1	CAGCTTCGTGAGCGACCTC	GGCAGTCGAGAAGTCCAGTC
mmu-MHC	GAGGGTGGCTCTCACACATTC	TTGGCCTTCGTAAGCAAACTG
mmu-Map1-lc3	CACTGCTCTGTCTTGTGTAGGTTG	TCGTTGTGCCTTTATTAGTGCATC
mmu-Atg7	GTTCGCCCCCTTTAATAGTGC	TGAACTCCAACGTCAAGCGG
mmu-Atg12	TCCGTGCCATCACATACACA	TAAGACTGCTGTGGGGCTGA
mmu-Beclin	TGAATGAGGATGACAGTGAGCA	CACCTGGTTCTCCACACTCTTG
mmu-Atg4b	ATTGCTGTGGGGTTTTTCTG	AACCCCAGGATTTTCAGAGG
mmu-Bnip3	TTCCACTAGCACCTTCTGATGA	GAACACCGCATTTACAGAACAA
mmu-Gabarapl1	CATCGTGGAGAAGGCTCCTA	ATACAGCTGGCCCATGGTAG
mmu-Cathepsinl	GTGGACTGTTCTCACGCTCAAG	TCCGTCCTTCGCTTCATAGG
mmu-Bnip3l	TTGGGGCATTTTACTAACCTTG	TGCAGGTGACTGGTGGTACTAA
mmu-Vps34	TGTCAGATGAGGAGGCTGTG	CCAGGCACGACGTAACTTCT
mmu-Murf2	AAAGCAACTGATCTGTCCCATC	TGTGGGTAAGTACGGGTTAGAG
mmu-Murf3	GGAGAAGCAGCTCATTTGCC	CCTCCTGAAGACACCGTTGTG
mmu-Mul1	CTGGGCACCAGTTCGATGG	GACAGCATAAGGCACACACTT
mmu-Traf6	AAAGCGAGAGATTCTTTCCCTG	ACTGGGGACAATTCACTAGAGC
mmu-Fbxo40	CGTCTCCTGCCTGGTGATAAG	GTATGCTCTGACTCTTTGCACAT
mmu-Znf216	CCCATGCTGTGTAGTACAGGA	GCTCATTCTGCCACTATTCTGC
mmu-Cblb	GGTCGCATTTTGGGGATTATTGA	TTTGGCACAGTCTTACCACTTT

mmu-Nedd4	TCGGAGGACGAGGTATGGG	GGTACGGATCAGCAGTGAACA
mmu-circDdb1	CAGTGCTTTGGGGTCCTTTC	CCTGAGGGTGGATCTGAAGG
mmu-Ddb1	GGCCTTCGACTCTACGATGG	GCAGTTCCTCTAGGCGGATG
mmu-circDdb1-Convergent	TGTCTCAAGAGCCCAAAGCT	TTCAATGCGGCTGGAAAGGA
mmu-circDdb1-Divergent	CAGTGCTTTGGGGTCCTTTC	CCTGAGGGTGGATCTGAAGG
hsa-circDdb1	CCAGCGCTAGCACCCA	CAGGAGGCCACACTGATGTT
mmu-eif4a3	TTTACGGACACGGGCTAT	TCACCAAGATGCGGATG

Table S3. The position of the specific fragment detected by mass spectrometry on Ddb1-867aa

circRNA specific ORFs	Length	Amino acid sequence
circRNA-Ddb1 Peptides	2601	MEHEVACLDITPLGDSNGLSPLCAIGLWTDI<br>SARILKLPSFELLHKEMLGGEIIPRSILMTTFE<br>SSHYLLCALGDGALFYFGLNIETGLLSDRKK<br>VTLGTQPTVLRTFRSLSTTNVFACSDRPTVIY<br>SSNHKLVFSNVNLKEVNYMCPLNSDGYPDS<br>LALANNSTLTIGTIDEIQKLHIRTVPLYESPRK<br>ICYQEVSQCFGVLSSRIEVQDTSGGTTALRPS<br>ASTQITSASVRLVSQEPKALVSEWKEPQAKN<br>ISVASCNSSQVVVAVGRALYYLQIHPQELRQI<br>SHTEMEHEVACLDITPLGDSNGLSPLCAIGL<br>WTDISARILKLPSFELLHKEMLGGEIIPRSIL<br>MTTFESSHYLLCALGDGALFYFGLNIETGLL<br>SDRKKVTLGTQPTVLRTFRSLSTTNVFACSD<br>RPTVIYSSNHKLVFSNVNLKEVNYMCPLNS<br>DGYPDSLALANNSTLTIGTIDEIQKLHIRTVP<br>LYESPRKICYQEVSQCFGVLSSRIEVQDTSG<br>GTTALRPSASTQITSASVRLVSQEPKALVSE<br>WKEPQAKNISVASCNSSQVVVAVGRALYYL<br>QIHPQELRQISHTEMEHEVACLDITPLGDSN<br>GLSPLCAIGLWTDISARILKLPSFELLHKEML<br>GGEIIPRSILMTTFESSHYLLCALGDGALFYF<br>GLNIETGLLSDRKKVTLGTQPTVLRTFRSLS<br>TTNVFACSDRPTVIYSSNHKLVFSNVNLKEV<br>NYMCPLNSDGYPDSLALANNSTLTIGTIDEI<br>QKLHIRTVPLYESPRKICYQEVSQCFGVLSSR<br>IEVQDTSGGTTALRPSASTQITSASVRLVSQE<br>PKALVSEWKEPQAKNISVASCNSSQVVVAV<br>GRALYYLQIHPQELRQISHTE
